# Thermal Imaging for Quality Control in Thin Silicon‐Based Coatings for Lithium‐Ion Batteries: Defect Detection, Drying Dynamics, and Machine Learning‐Based Mass Loading Estimation

**DOI:** 10.1002/smtd.202402079

**Published:** 2025-04-14

**Authors:** Adil Amin, Philipp Valentin Geiping, Ahammed Suhail Odungat, Fatih Özcan, Doris Segets

**Affiliations:** ^1^ Institute for Energy and Materials Processes–Particle Science and Technology (EMPI‐PST) Carl‐Benz‐Straße 199 47057 Duisburg Germany; ^2^ Center for Nanointegration Duisburg‐Essen (CENIDE) University of Duisburg‐Essen (UDE) Carl‐Benz‐Straße 199 47057 Duisburg Germany

**Keywords:** battery electrode, defect detection, drying dynamics, non‐destructive testing, quality control tool, silicon‐based thin coatings, thermal imaging

## Abstract

This study demonstrates thermal imaging as a non‐destructive, real‐time quality‐control‐method for detecting coating defects, analyzing mass loading, and understanding drying dynamics in silicon‐based thin coatings. Thermal imaging identifies critical defects such as streaks, pinholes, and chatter marks through distinct thermal signatures, with streaks reducing surface temperature by up to 15 °C. It establishes strong correlations between surface temperature, mass loading, and coating thickness: for instance, a 100 µm wet film thickness shows a surface temperature of ≈50 °C, corresponding to a mass loading of 2.4 mg cm⁻^2^. Drying dynamics reveal that thicker coatings retain more solvent, prolong drying, and shrink significantly, with 100 µm wet‐gap coatings shrinking by up to 60%. A Random Forest machine learning model predicts mass loading with high accuracy (±0.3 mg cm⁻^2^) using surface temperature data, highlighting the feasibility of thermal imaging‐based quality estimation. While validated in a batch process, this approach is well‐suited for integration into roll‐to‐roll production across diverse thin coating applications, such as batteries, solar cells, and functional films. Thermal imaging provides a robust pathway for real‐time defect detection, drying optimization, and quality control, improving coating performance and production reliability.

## Graphical abstract

Thermal imaging offers a non‐destructive approach to quality control in silicon‐based lithium‐ion battery electrodes, enabling the detection of defects, variations in mass loading, and the monitoring of drying dynamics. This study introduces an automated defect‐detection‐algorithm and a machine learning‐based Random Forest model to estimate mass loading from thermal imaging data. While applied in a batch process, these methods could be adapted for inline quality control in future studies.

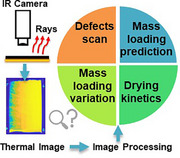

## 1 Introduction

The rapid growth in the production capacity of lithium‐ion batteries (LIBs) is driven by the global transition to renewable energy and the increasing demand for electric vehicles.^[^ ^1^ ^]^ Beyond its material properties, the performance of a LIB is fundamentally influenced by the uniformity and quality of its electrode coatings.^[^ ^2^, ^3^ ^]^ Electrode coating should be homogenous and defect‐free. There is a huge number of defects that can occur during coating and these must be minimized, e.g., uneven material distribution, agglomerates, divots, pinholes, streaks, contaminations, cracks, or variations in thickness and weight.^[^ ^4^ ^]^ The presence of defects can compromise electrode performance, leading to reduced capacity, decreased cycling stability, lower safety, or premature failure.^[^ ^5^, ^6^ ^]^ The scrap rate in LIB electrode manufacturing is estimated at ≈2%,^[^ ^7^ ^]^ contributing to ≈6% of the overall battery cost.^[^ ^8^ ^]^ As production scales up in gigafactories, this impact becomes more significant, emphasizing the need for improved quality control methods. High‐resolution imaging methods such as micro‐computed tomography are widely used for detailed defect characterization, allowing for an in‐depth analysis of internal structures, porosity, and layers. It is particularly valuable for analyzing fully assembled battery cells.^[^ ^9^ ^]^ However, for large‐scale manufacturing, real‐time inline testing methods remain important for rapid defect detection and thickness/coat weight measurement, to enhance coating uniformity and reduce waste.^[^ ^2^, ^10^ ^−^ ^12^ ^]^ Inline process monitoring is important for accurate modeling, enabling data collection for digital twins that predict outcomes, optimize manufacturing, and support inline control algorithms.

The current state‐of‐the‐art quality control for LIB electrodes primarily relies on inline defect detection using photo‐optical camera systems, while thickness and/or mass loading are typically measured through laser calipers and beta transmission, respectively.^[^ ^13^, ^14^ ^]^ A photo‐optical camera system effectively identifies large surface defects such as cracks and streaks,^[^ ^15^ ^]^ whereas laser calipers provide precise thickness measurements.^[^ ^16^ ^]^ Beta transmission, which utilizes the attenuation of beta radiation, enables simultaneous assessment of coating thickness and mass loading. However, these conventional techniques have inherent limitations. Detecting small defects such as pinholes, agglomerates, divots, contaminants, and blisters using an optical camera is challenging due to the rough and dark surface of the coating, as well as substrate reflectance, both of which generate significant optical noise that can obscure small defects.^[^ ^10^ ^]^ Additionally, laser calipers require precise alignment, making traversal along the moving web more challenging. They provide point‐ or line‐based readings, potentially missing localized defects or coating inconsistencies.^[^ ^16^ ^]^ Furthermore, the beta transmission measurement is an expensive technique and poses environmental hazards due to ionizing radiation.^[^ ^16^, ^17^ ^]^ Given the limitations of conventional quality‐control‐methods, there is an increasing interest in exploring complementary non‐destructive techniques to enhance electrode inspection and create a more robust detection framework.^[^ ^16^ ^]^ Among these, thermal imaging has gained attention as a promising approach. It can complement existing methods and address their limitations. However, its potential in LIB battery electrode manufacturing remains an area of active investigation.

Thermal imaging is a non‐contact inspection technique based on the physical principle that any object with a temperature above absolute zero (0 K) emits electromagnetic radiation. The intensity and spectral composition of this radiation are directly correlated with the object's surface temperature and properties.^[^ ^18^ ^]^ By capturing this infrared radiation (IR), thermal imaging generates a temperature map that reveals variations across the surface (in our case the surface of electrodes).^[^ ^14^, ^19^ ^]^ Thermal imaging techniques can be broadly categorized into steady‐state thermography,^[^ ^20^ ^]^ which relies on continuous heating to maintain a stable thermal gradient, and transient thermography, which involves applying a short‐duration heat pulse^[^ ^21^, ^22^ ^]^ or modulated heating^[^ ^22^, ^23^ ^]^ to analyze the material's thermal response over time. Steady‐state thermography offers a practical advantage for roll‐to‐roll (R2R) manufacturing due to its continuous heating, making it well‐suited for real‐time monitoring of large‐area coatings without requiring complex synchronization. While its sensitivity to subsurface defects may be lower compared to transient methods, certain anomalies such as voids, delamination, or embedded contaminants can still be indirectly detected if they alter heat conduction and cause measurable variations in surface temperature. In contrast, transient thermography, including pulse and lock‐in techniques, provides superior sensitivity to subsurface defects by analyzing dynamic thermal responses.^[^ ^22^, ^24^ ^]^ While effective for depth‐resolved imaging, transient thermography could face challenges in integration into an R2R process due to the need for precise timing, controlled heat excitation, and longer acquisition times, making it less adaptable for high‐speed production lines.

At this point, it has to be mentioned that thermal imaging of coating surfaces is not a new technique. Historically, it has primarily been used for non‐quantitative imaging, often limited to detecting defects such as debonds on Thermal Barrier (TB) coatings, which are ceramic‐based protective layers used in aerospace and industrial applications to provide thermal insulation.^[^ ^25^ ^]^ In 2011, researchers at GE company reported its first use for imaging the full‐area coating thickness in such applications (TB coatings).^[^ ^26^ ^]^ The first documented use of thermal imaging for defect detection in LIB electrode production appeared in a study from the year 2013 on infrared particle detection for battery electrode foils, where researchers used thermal imaging to identify contaminations from slivers of aluminum and copper in carbon electrodes.^[^ ^14^ ^]^ A closely related study by Nathan Sharp and colleagues in 2014 further explored this approach, employing pulse thermography to evaluate LIB electrode quality (cathode: LiCoO_2_), successfully detecting defects such as oil impurities, scratches, thickness variations, and composition inconsistencies.^[^ ^27^ ^]^ Later on, some studies reported the use of thermal imaging for defect detection in LIB electrode coatings during production.^[^ ^2^, ^16^ ^]^ However, its application for measuring electrode coating (specifically for next‐generation Si‐based anode) thickness, mass loading, and drying behavior in LIBs remains underexplored and an area of active research.

In this study, we used thermal imaging (steady‐state) in a batch process to analyze laboratory‐scale thin coatings of silicon/carbon (Si/C) composite nanoparticles, beginning with its application for identifying and classifying three major defects: streaks, pinholes, and chatter marks. This demonstrates the potential of the method for defect detection and quantification. To enable a systematic investigation, we also established methods to controllably generate these defects, providing a reproducible approach for future studies examining their effects on coating quality and performance. We explored variations in mass loading and coating thickness, highlighting how thermal imaging can capture changes in these critical parameters. We also demonstrated the potential, as an initial proof of principle, to model the estimation of mass loading and coating thickness using surface temperature data of thermal images. Finally, we explored the drying process of the electrodes to understand their dynamic behavior. Together, our study reveals the capability of thermal imaging as an effective approach to monitor essential parameters in LIB electrode manufacturing, while also building knowledge that could potentially be extended to other electrode materials and applications.

## 2 Methods Development

### 2.1 Materials

Si/C composite nanoparticles, produced via a gas‐phase process as described elsewhere,^[^ ^28^ ^]^ were selected for this thermal imaging study due to silicon's critical importance as a next‐generation anode material in batteries and its role as an active material in solar cells. Si/C nanoparticles (Figure S1, Supporting Information) were in the form of partly sintered aggregates, with primary particle sizes ranging from 100 to 250 nm and average aggregate sizes of ≈550 nm.

### 2.2 Characterizations

The morphology and primary particle sizes of the Si/C active material were characterized using a JEM‐2200FS (JEOL, Japan) transmission electron microscope, operating at an accelerating voltage of 200 kV. Surface temperature distribution across the coated electrodes was monitored using a TIMQVGA‐HD O29 thermal imaging camera/IR‐camera (Micro‐Epsilon Messtechnik GmbH & Co. KG, Germany). Flatbed photo scanner (Cannon Scanner K10485, Vietnam) was used to capture high‐resolution images of the dried coated layers, enabling visual inspection and comparison with thermal camera images. The mass of each electrode was determined using a high‐precision analytical balance (KVD), and the coating thickness of the dried electrodes was measured with a digital micrometer screw gauge (Pollin Electronic Ladengeschäft, Germany).

### 2.3 Experimental

#### 2.3.1 Slurry Making, Coating and Drying Protocol

Slurries with a total solid content of 30 wt.%, consisted of Si/C active material, carbon black, and poly(acrylic) acid in a solid mass ratio of 80:5:15. All slurries were prepared using a two‐step dispersion process described in our previous work (Figure S2, Supporting Information).^[^ ^29^ ^]^ The slurries were applied onto an 18 µm thick copper foil (Schlenk, Germany) using a blade coater (TQC Sheen, Industrial Physics, Netherlands) at a coating speed of 10 mm s^−1^. The wet coating thickness was controlled by adjusting the doctor blade gap, called wet‐gap height. For the analysis of as‐prepared electrodes, the coated electrodes were dried in an oven at 80 °C for 1 h under atmospheric pressure before they were transferred to the heated bed for thermal imaging.

To ensure clarity, we define two key terms used throughout this work. The wet‐gap height, also referred to as wet‐layer thickness, corresponds to the initial thickness of the coating immediately after the spread of the slurry on the copper foil substrate, before the drying process begins. This thickness is determined by the gap setting of the coating tool. In contrast, the dry layer thickness refers to the final thickness of the electrode coating after the solvent has fully evaporated during the drying process. As a result of solvent loss and material shrinkage, the dry layer thickness is typically significantly lower than the wet‐layer thickness.

#### 2.3.2 Defects Generation

To systematically investigate defect detection using thermal imaging, we designed artificial defects to mimic commonly observed coating irregularities, ensuring that their shapes and patterns closely resemble real manufacturing defects (Table S1, Supporting Information) as observed and reported by various researchers.^[^ ^2^, ^11^, ^12^, ^30^ ^]^ To generate such defects in our study, artificial model systems were developed. Streaks were created by scratching the wet coatings with a spatula, producing elongated marks parallel to the coating direction. Chatter defects, simulating vibrations, were generated by placing strips of adhesive tape under the copper foil (Figure S3, Supporting Information), resulting in periodic lines due to rhythmic vibration of the doctor blade perpendicular to the coating direction. Pinholes were generated by air entrapment in the slurry during its preparation: the lid of the slurry mixing tube was left open while mixing at a high speed of 4000 RPM, allowing air to be dragged into the slurry. When this slurry was coated onto the copper substrate, air‐bubble‐bursting during drying led to pinhole defects.

The artificial defects were created with controlled dimensions and spacing to ensure reproducibility, allowing for a systematic study of their influence on thermal contrast and detectability. However, pinhole defects serve as a pseudo‐real scenario, as air entrapment is a well‐known issue in slurries and commonly causes such defects. Unlike streaks and chatter marks, which were intentionally introduced by our intervention, pinholes were generated more naturally from environmental and process‐related factors rather than direct human intervention.

In conclusion, by generating these defects in a controlled manner, we established a reproducible testbed for evaluating defect detection and characterization in thin silicon‐based coatings. Generating these controlled, repeatable defects allowed us to validate the thermal camera's potential as a quality control tool when it is combined with image analysis.

#### 2.3.3 Measurement Set‐Up

**Figure 1:**
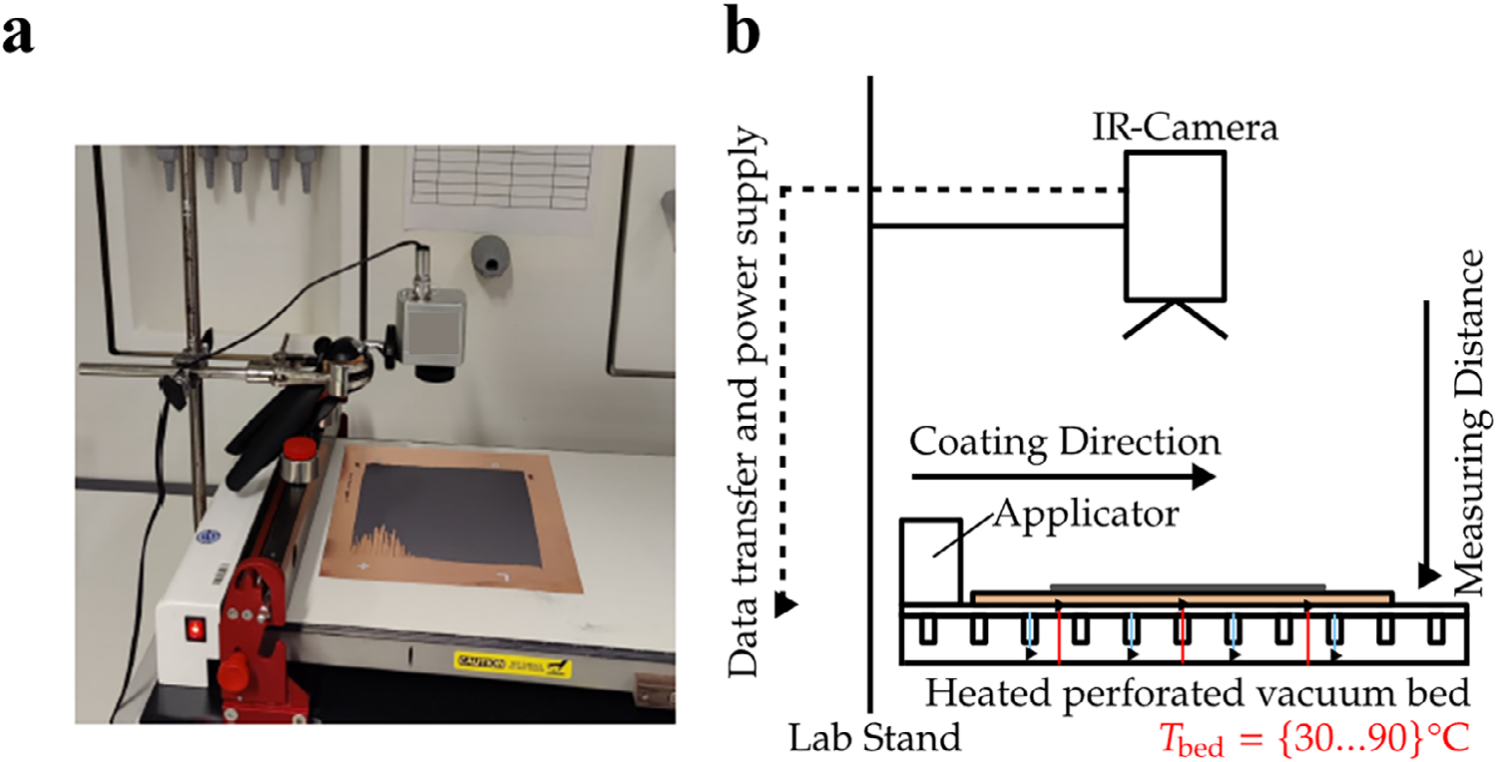
a) Photograph and b) schematic of the measuring setup, respectively.

To capture the surface temperature distribution of the coated electrodes, the thermal camera was positioned perpendicular to the sample, with an adjustable measuring distance of 12 to 40 cm (**Figure** **1**). The temperature range for detection was set from 20 to 100 °C, with an emissivity value of 0.9 for the coated surface, allowing for accurate detection of temperature variations. The TIM Connect software (Release 3.18.3103.0, Micro‐Epsilon) was used for data acquisition, and temperature values were recorded as. csv files for further processing. The raw thermal imaging data was saved as 382 × 288 matrices. Each element in the matrix corresponds to the surface temperature of a pixel (px) in the recorded image.

### 2.4 Data Processing and Statistics

#### 2.4.1 Image Processing

The information contained in thermal images, also called thermographs,^[^ ^19^ ^]^ can be organized into a matrix with 𝐾 rows and 𝑀 columns:

(1) $$T\left(x,y\right)=\left[\begin{array}{ccc} T_{11} & \text{…} & T_{1M}\\ \vdots & \ddots & \vdots \\ T_{K1} & \text{…} & T_{KM} \end{array}\right]\circ C$$

**Scheme 1:**
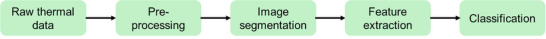
Processing flow for thermal image analysis.

Each element in the matrix corresponds to a temperature value recorded as a single px by the thermal camera. A time‐resolved series of thermal images of the same object can be viewed as a sequence of these matrices, *T* (x, y, t), where each matrix captures the temperature distribution across the object's surface at a specific time step. In this study, the thermal imaging data were processed using MATLAB (version R2024b, License No. 40 950 020, provided by the University of Duisburg‐Essen) with custom‐developed codes, following the method shown in **Scheme** **1**.

The image processing techniques presented below will be applied to analyze coated anode layers, with the goal of identifying and classifying common coating defects and identifying mass loading/thickness variations in battery electrode fabrication.

*Pre‐processing*: Once imported, the raw data was pre‐processed to minimize noise and prepare it for further analysis. Various filters were applied based on the specific objective, such as noise reduction, edge detection, or enhancing image quality. The filters used included discrete approximations of Gaussian and median filters. The Gaussian filter, represented by a 3 × 3 kernel, was convolved with each px in the thermal image, effectively smoothing the data by reducing random noise.

(2) $$F_{gauss}\left(x,y\right)=\frac{1}{16}\left[\begin{array}{ccc} 1 & 2 & 1\\ 2 & 4 & 2\\ 1 & 2 & 1 \end{array}\right]$$

*Image segmentation*: Image segmentation involves dividing an image into distinct regions, with thresholding being one of the simplest methods for this purpose. During thresholding, each px is evaluated against a set threshold value and assigned to a binary image. Px with values below the threshold are given a value of zero, while those above it receive a value of one. In this work, thresholding was used to create masks that differentiate between regions containing the coating and areas displaying the copper substrate.

*Feature extraction*: Feature extraction refers to the transformation of data to emphasize specific image regions containing relevant characteristics. For defect detection, regions with temperature irregularities can be highlighted. One tool commonly used for this purpose is the Laplacian of Gaussian (LoG) filter. Here, the second derivatives were calculated in both image directions based on an image adjusted using the Gaussian filter.

(3) $$F_{LoG}\left(x,y\right)=\Delta F_{gauss}\left(x,y\right)=\frac{\partial^{2}F_{gauss}\left(x,y\right)}{\partial x^{2}}+\frac{\partial^{2}F_{gauss}\left(x,y\right)}{\partial y^{2}}$$

A discrete representation of this filter, in the form of a kernel, was convolved with the image on a px‐by‐px basis, particularly emphasizing areas with steep temperature gradients.

The second filter type used in this work was the “dimensionality filter” for filtering streak defects based on their characteristic shape. Regions were classified as streaks if their length parallel to the coating direction was at least twice their elongation in the perpendicular direction. This ensured the effective differentiation of streak defects from other types of defects.

The third filter type used in this work was a Sobel filter. The Sobel filter is a type of edge‐detection filter used to highlight regions of high‐intensity change in an image, effectively emphasizing edges. It works by calculating the gradient at each px, typically in horizontal or vertical directions, to detect boundaries or linear features. The Sobel filter is widely used in image processing to reveal structural details and enhance contrast along edges. In this work, a Sobel filter was applied to the thermal image data to emphasize vertical lines (chatter defect type). This filter calculated the gradient of image intensity at each px, highlighting vertical edges or temperature transitions. This approach proved useful in detecting linear features and boundaries within the images, enhancing the clarity of relevant structures for analysis.

Hit‐and‐Miss operation is a morphological operation used for refining feature extraction in binary images. It is a pattern‐matching tool that refines feature detection by identifying specific shapes while ignoring others. In this context, it serves as a secondary filter to remove small, insignificant features that might have been highlighted by the Sobel filter but are not relevant to the analysis. This operation helps to focus on only the significant vertical features by filtering out minor noise or irrelevant details, thus refining the detection and making the results more accurate.

*Classification*: Once Regions of Interest (ROIs) were detected in the image, classification methods, including supervised and unsupervised approaches, were applied to categorize the regions based on different defect types. Specific geometrical constraints, such as aspect ratio and the alignment of the ROI relative to the coating direction, were used to assist in this classification.

*Image registration*: Image registration is a technique for aligning two images within a common coordinate system. It is especially useful when images have distortions, translations, or scale differences, such as those captured by different measurement instruments. Although image registration is widely known and commonly used in fields like medical imaging, we developed a custom approach incorporating specific reference frames and code to achieve precise overlay by detecting distinct markers.

In this work, we used the Maximally Stable Extremal Regions (MSER) algorithm to overlay thermal camera images with digital scans of dried layers. Coated layers were marked with symbols drawn with a white metallic lacquer pen, and 12 mm‐diameter circular electrodes were then punched from these coatings for weighing and mass loading. After stamping, the coatings were digitally scanned using a flatbed scanner. These markers, visible in both thermal and scanned images, enabled accurate alignment. To precisely match surface temperatures from thermal images with the corresponding punched electrode weights, the MSER algorithm was applied to extract the markings, ensuring an accurate overlay of thermal images with scanned images.

#### 2.4.2 Sample Sizes and Standard Deviation

For experiments investigating the relationship between mass loading, thickness, and surface temperature data of the coating, results are presented as mean ± standard deviation. Sample sizes varied depending on the specific experiment. For coatings produced using an inclined doctor blade, where the gap between the blade and substrate gradually increases (referred to as wet‐gap height variation), the coated region at each condition was divided into three sections. From each section, six circular electrodes with a diameter of 12 mm were selected, resulting in a total of 18 electrodes per condition.

For coatings prepared with fixed wet‐gap heights, a total of 18 electrodes from a whole coating were analyzed per experimental condition to evaluate the correlation between mass loading, coating thickness, and surface temperature under uniform coating conditions. In this analysis, mass loading values correspond to individual electrodes, while surface temperature values represent the mean temperature across the surface of each 12 mm‐diameter electrode. The standard deviation reflects local temperature variations within each electrode.

For the estimation of the mass loading (mg cm^−2^) in a specific region of the coating using surface temperature data, predictive models were developed. For model development, mass loading and surface temperature data from 90 electrodes (18 electrodes per uniform coating condition) were used to establish fitting equations for conventional regression models (piecewise, polynomial, and logarithmic fits) and to train the Random Forest machine learning model. For model validation, an additional 36 electrodes from a new coating were used to independently assess prediction performance. Model accuracy was evaluated by comparing predicted mass loading values with actual measurements obtained from electrode weighing. The R^2^ value was used to evaluate the goodness of fit, while the Mean Absolute Error (MAE) and the Root Mean Squared Error (RMSE) quantified the prediction error.

#### 2.4.3 Predictive Modeling: Conventional and Machine Learning Approaches

To develop predictive models for estimating mass loading from surface temperature data, both traditional regression techniques and amachine learning method were employed. Traditional regression models, including piecewise, polynomial, and logarithmic fits, were used for their simplicity and interpretability. The piecewise model divides the dataset into segments, fitting separate functions to different regions, making it effective for capturing abrupt changes in trends.^[^ ^31^ ^]^ The polynomial model represents nonlinear relationships by fitting a polynomial function to the data. Including more polynomial terms improves flexibility but also raises the risk of overfitting.^[^ ^32^ ^]^ The logarithmic model (e.g., *y*  =  *a*log (*x*) +  *b*), though being fully empirical, is particularly suited for data that initially exhibits rapid changes but stabilizes over time.^[^ ^33^ ^]^ These regression models help understand the dataset and act as baseline predictors alongside the more advanced machine learning model.

A Random Forest^[^ ^34^ ^]^ is an ensemble learning method that constructs multiple decision trees using random subsets of the training data. Each tree is built by considering random subsets of features at each decision split, and predictions from each tree are aggregated (averaged for regression or majority voting for classification) to form the final prediction. In this study, we used the Random Forest regressor from the scikit‐learn^[^ ^35^ ^]^ library (version 1.5.1) in Python to model the relationship between surface temperature and mass loading of electrodes. The scikit‐learn library is designed for various machine learning tasks such as classification, regression, and clustering. The data was split into training and pre‐testing sets (80% for training and 20% for pre‐testing), and the model was trained using 100 decision trees. The model was saved using joblib for future use, enabling easy predictions on new data.

## 3 Applications

### 3.1 Defect Detection in Coated Electrodes: Method Application and Analysis

As a first step, we introduced artificial defects, such as streaks, chatter, and pinholes, to demonstrate the effectiveness of thermal imaging in detecting these common flaws, which frequently arise during the coating stage. We chose to use artificial defects because they provide consistency and control, enabling us to reliably simulate real‐world conditions. Detecting these defects is critical due to their significant negative impact on the electrochemical performance of electrodes within cells and the additional safety risks they pose.

As previously mentioned, the detection of these defects is important during manufacturing as it can be detrimental to the performance of the electrode. For example, Mohanty et al.^[^ ^5^ ^]^ studied the effect of electrode manufacturing defects in NMC532 cathodes on LIB performance. They found that agglomerates caused a rapid capacity fade, with only 12% retention at 2C and 14% at 5C after 200 cycles, compared to 70% and 50% for the baseline, respectively. Pinholes showed less severe capacity fading, retaining 47% at 2C and 40% at 5C. Moreover, the defect consisting of three parallel streaks exhibited significantly worse performance, retaining only 7% capacity. Additionally, electrodes consisting of one single streak defect performed better than electrodes with three streak defects but still showed reduced capacity retention, holding 45% after 200 cycles at 5C, compared to the value of 50% that was exhibited by the baseline.

**Figure 2:**
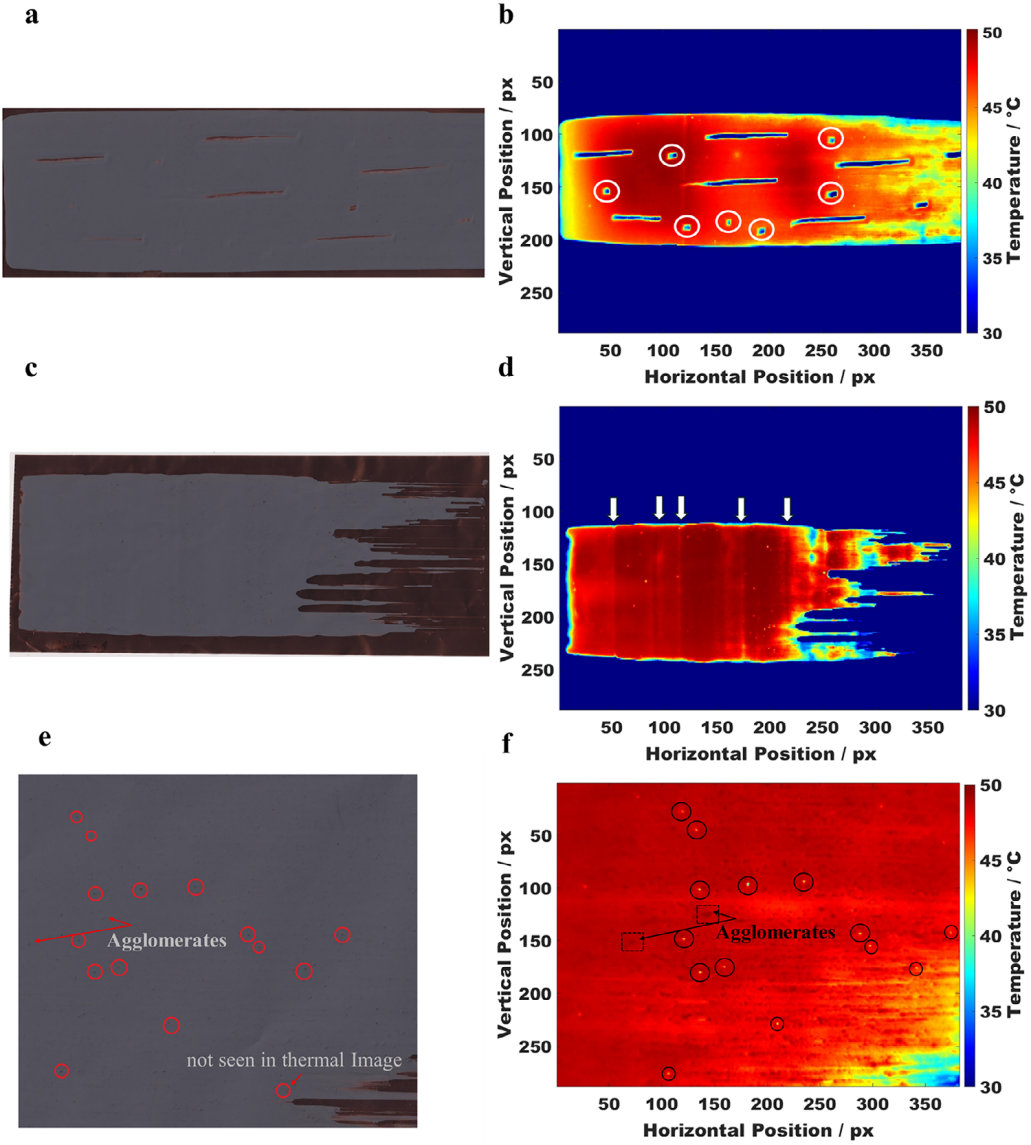
Photo scans of test coatings showing defect types: a) streaks, c) chatter, and e) pinholes (close up), respectively, and corresponding thermal images: b) streak defects, d) chatter defects (arrows indicate regions with 5 °C lower temperatures compared to adjacent smoothly coated areas), and f) pinhole defects, captured as a close‐up from a 120 mm measuring distance. Note: Photo scan of the full coating with pinhole defects can be seen in Figure S5 (Supporting Information).

**Figure** **2**a shows a digital scan image of the test coating captured using flatbed scanner for digital photos, while Figure 2b presents the thermal camera image detecting the streaks. In all the detected streaks, a reduction in the surface temperature from 40 °C down to 25 °C is visible. Furthermore, the streaks appear as valleys with higher temperatures on the edges which decrease inwards. The digital scan of the coated layer shows how slurry has accumulated at the edges of the streak because of scraping with the spatula. However, certain regions in the thermal image could not be classified as streak defects (at coordinates: 50, 150 px; 125, 125 px; 125, 190 px; 175, 180 px; 200, 190 px; 275, 140 px; 275, 100 px, highlighted with white circles in Figure 2b). To calculate the total area comprising streak defects, a dimensionality filter was applied to the thermal image (Figure 2b). Regions not meeting the aspect‐ratio‐criterion, where the length parallel to the coating direction is at least twice the perpendicular elongation, were excluded. Only regions elongated along the coating direction (left to right) were considered, leveraging the characteristic geometry of streaks. In the example shown (Figure S4, Supporting Information), 1.94% of the total coating area was identified as streak defects.

Figure 2c shows a photo scan of the test coating and the isolated thermal image of the respective coating to show the detection of chatter defects by thermal imaging (Figure 2d). In thermal images, these defects appeared as periodic temperature variations across the surface, that were detectable by using image segmentation techniques. In chatter defects, the temperature drops by 5 °C than the maximum temperature of smoothly coated areas. Chatter defects could be filtered solely by applying the Sobel filter to the thermal image data to highlight vertical lines, and a hit‐and‐miss operation further refined the detection by excluding small or insignificant features (Figure S6, Supporting Information). In the shown test coating, a total of 2.38% of the area is considered as chatter defects. This is particularly important as in contrast to streak defects, these types of defects are hard to detect by eye from scanned images.

Finally, Figure 2e,f shows a scan of the coated layer with pinhole defects (close up) and the corresponding thermal image of the layer respectively. In the raw thermal image (Figure 2f), small regions with lower temperature values (close to 40 °C) than the surrounding coating indicated the presence of pinholes. The largest pinhole defect, located at position (180, 100 px), occupied a measurable area of 4 × 4 px, making it detectable within the image. The automatic detection, processed through MATLAB, successfully filtered most of the pinhole defects, as shown in Figure S7 (Supporting Information). However, defects at horizontal positions ≈140 px and ≈290 px, which were visible in the scan (Figure S5, Supporting Information), were not detected automatically. This limitation stemmed from the geometric filtering applied during detection. The filter checks the circularity of each ROI, and due to the resolution limit of the thermal camera, defects were detected down to the level of single px. If the geometry of the ROI deviated from a circular shape, such as regions measuring 1 × 3 px, it was excluded from the pinhole classification. Overall, 0.00098% of the total image area was identified as pinhole defects, highlighting the precision of the detection method. This quantification evidences the capability of thermal imaging for detecting even small‐scale defects in coated electrodes, with room for refinement in addressing limitations related to resolution and geometric filtering.

Therefore, it can be stated that using image processing, various defect types were successfully detected and classified. However, a compromise between field of view and spatial resolution was observed: moving the thermal camera closer improved the spatial resolution for smaller defects like pinholes, agglomerates, and heat cracks. But at the same time, it also reduced the field of view, causing some defects outside the observed area to remain undetectable. To aid reader comprehension, a schematic showing this limitation is provided in Figure S8 (Supporting Information). This limitation, inherent to the camera's resolution, can be mitigated by using a high‐resolution thermal camera, which would enable the detection of small defects without reducing the field of view, ensuring that all defects remain visible.

Close‐ups also allowed the detection of additional defects, such as agglomerates (for example, at 150, 125 px; 75, 150 px) and shallow streaks (for example, 250, 180 px). Finally, it needs to be mentioned that in addition to pinhole defects, the thermal image (Figure 2f) revealed surface heterogeneity, with circular hotspots. These spots showing higher surface temperatures compared to the neighboring regions could indicate local variations in the active material and carbon black mixture, for instance, due to agglomeration or inequal mass loading. This will be discussed in the following.

### 3.2 Mass Loading and Thickness Variations

#### 3.2.1 Sample Coatings with Wet‐Gap Height Gradients

As an initial approach to assess whether variations in mass loading and thickness can be detected using thermal imaging, model systems were coated with inclined blades to create controlled gradients of mass loading/thickness. To achieve these gradients, we adjusted the doctor blade by setting one side to a higher gap and the other to a lower gap, effectively controlling the wet‐layer thickness across the coating. By controlling the wet‐layer thickness in this way, we generated coatings with a gradual dry‐thickness gradient after drying.

By preparing these coating samples using specific gradient wet‐gap settings (100–20 µm, 100–40 µm, 100–90 µm, and a constant 100 µm wet‐gap), we systematically examined how variations in coating thickness influenced the thermal response. Similar to the previous investigation of model defects, this setup allowed us to explore the sensitivity and limits of thermal imaging in detecting differences in mass loading and thickness, providing valuable insight into its effectiveness for assessing coating uniformity.

**Figure 3:**
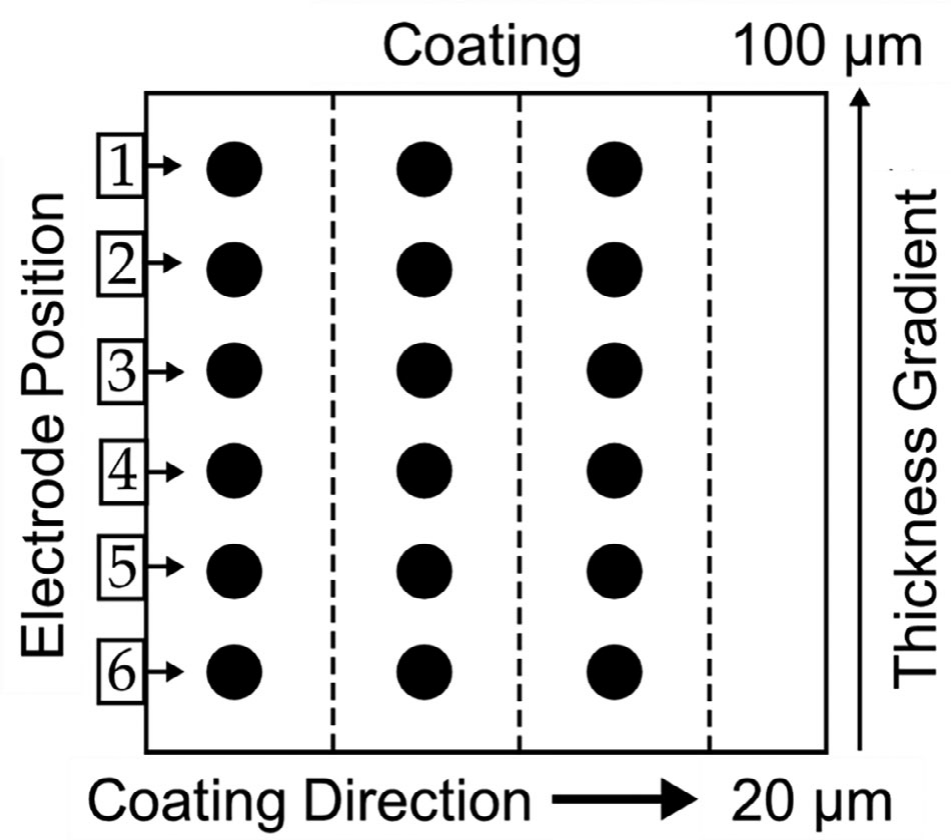
Positions of electrodes punched from samples coated with an inclined doctor blade. The coating is divided into three regions, each containing six electrodes punched perpendicular to the direction of the coating.

To ensure accuracy and reliability, temperature data were extracted from three equidistant line graphs (see **Figure** **3**) across each of the four samples, including the constant 100 µm sample, with average values and standard deviations calculated for each. This statistical analysis across all samples quantifies temperature variations and identifies detectable thresholds for thickness and mass loading variations. Additionally, coin‐shaped electrodes were punched from three different regions, with six electrodes per region, resulting in a total of 18 electrodes per sample. These electrodes were used to gather areal mass loading data, which was then compared with the corresponding surface temperatures. The method used to calculate mass loading and dry layer thickness is detailed in SI Section S4.7 (Supporting Information). This comparison is essential to validate whether temperature measurements can effectively represent the mass distribution, enabling us to further refine thermal imaging techniques for coating quality assessment.

**Figure 4:**
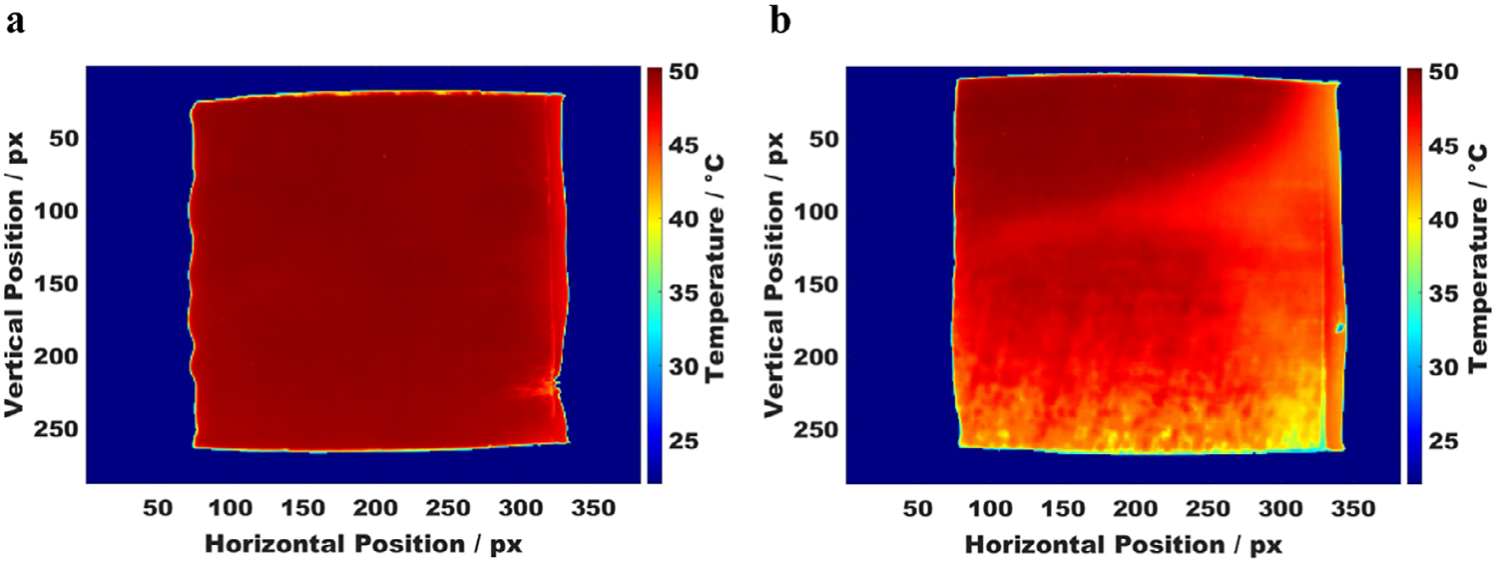
Thermal images of coatings prepared with different doctor blade wet‐gap settings. a) Reference thermograph with a consistent 100–100 µm blade wet‐gap, showing minimal temperature variations across the surface. b) Thermograph of a coating applied with an inclined blade setting of 100–20 µm, displaying a noticeable temperature gradient from top to bottom, as indicated by the color transition from dark red to yellow and green along the bottom boundary. The coating direction was from left to right.

**Figure** **4**a shows the reference thermograph for a coating applied with a consistent doctor blade gap of 100–100 µm, set to achieve a wet‐layer thickness of 100 µm along the length of the blade. This uniform gap setting results in minimal temperature variation across the surface. Although there was no gradient in the wet layer, slight temperature drops (color intensity change from dark red to red) were observed across the coated layer. Specifically, the temperature decreased by ≈1–1.5 °C, with one side of the coated layer showing an average temperature of 50 °C (matching the set bed temperature) and the other side being 1–1.5 °C lower. This variation may be attributed to differences in mass loading or coating thickness, likely influenced by the rheological behavior of the nanoparticle‐based slurry. In our previous publication,^[^ ^36^ ^]^ we demonstrated that nanoparticle slurries often result in coatings with uncontrolled mass loading and porosity/thickness. However, this study focuses solely on layer thickness and mass loading, not porosity. Thus, even with non‐inclined blades, we expect slight variations in mass loading and dry layer thickness due to the intrinsic properties of nanoparticle‐based slurries.

In contrast, Figure 4b shows a thermograph of a coating prepared with an inclined doctor blade setting of 100–20 µm, resulting in a noticeable temperature gradient across the coated layer. This gradient is evident in the change in color intensity, shifting from dark red at the top (near the 100 µm setting) to light red and eventually to green toward the bottom (near the 20 µm setting). This color transition reflects the gradual decrease in surface temperature, with higher temperatures near the thicker regions and lower temperatures in the thinner areas. The temperature starts around 50 °C near the top, matching the set bed temperature, and gradually decreases to a minimum of ≈42.5 °C at the bottom. This observed temperature gradient likely results from variations in coating thickness and mass loading caused by the inclined blade. The thicker sections near the 100 µm setting exhibit higher surface temperatures due to increased material mass, which emits more IR radiation. Conversely, the thinner areas near the 20 µm setting emit less IR radiation, resulting in lower detected surface temperatures.

These temperature variations highlight the sensitivity of thermal imaging in detecting subtle differences in coating thickness and mass distribution. This capability is particularly valuable for quality control in coatings that require uniformity in height and mass loading. Additionally, for applications where coatings are designed with precise gradient specifications, thermal imaging enables accurate monitoring of spatial temperature variations associated with thickness and mass distribution.

**Figure 5:**
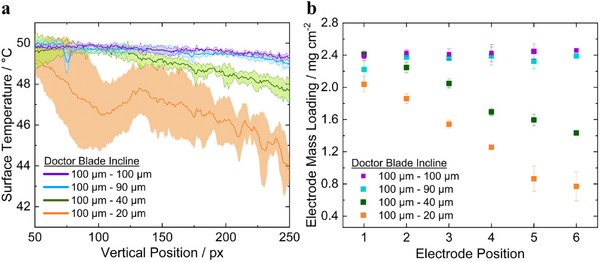
a) Line graphs showing the temperature decrease on the dried‐coating surface with decreasing blade gap height. Three line graphs were averaged, with the standard deviation represented by the filled area. Each line graph includes measurements from six electrodes, totaling 18 electrodes per sample. b) Measured electrode mass loadings (mg cm^−2^) at different vertical positions along the inclined coating, where the position increases from the left side of the doctor blade (set at a larger gap height of 100 µm) to the right side, where the gap height progressively decreases. Each data point represents the average of three electrodes, with the standard deviation shown as error bars. For visualization of electrode positioning, refer to Figure 3.

To be more quantitative, **Figure** **5** summarizes the plotted relationship between specific gradient wet‐gap height settings of the doctor blade (100–20, 100–40, 100–90, and a constant 100 µm wet‐gap) and two critical parameters: electrode mass loading and surface temperature of the corresponding coating across the coating width. The purpose of this figure is to examine how variations in wet‐gap height influence these parameters, providing insight into the impact of the blade's inclination on the overall coating quality. From a quality control perspective, this figure shows how changes in mass loading due to blade inclination led to variations in the surface temperature of the coating, which are then captured using thermal imaging.

Specifically, Figure 5a plots the coatings surface temperature profile for each gradient wet‐gap height setting. It can be seen from the figure that the coating's surface temperature decreases where smaller wet‐gap heights of blade are set. This plot is based on the average of three temperature line graphs along the width of the coating at different positions of the coating length (130, 220, and 250 px), with the filled area representing the standard deviation. Figure 5b, on the other hand, presents the measured electrode mass loadings (in mg cm⁻^2^) at different blade inclinations along the coating, with the positions ranging from 100 µm on the left side of the blade to varied gap heights on the right.

As shown in Figure 5a, for all four samples, the surface temperature near the 100 µm gap height region of the doctor blade remained around 50 °C. It was observed that both surface temperature and electrode mass declined more sharply as the gap height gradient increased.

For the 100–20 µm gradient, the surface temperature decline reached 11%, correlating with a 62% reduction in electrode weight. Similarly, for the sample with a 100–40 µm gradient, the surface temperature dropped by 3.8% corresponding to a reduction in mass loading by 40%. The other samples displayed lesser declines in surface temperatures: 1.7% for the sample with a 100–90 µm gradient and 1.2% for the sample with a 100–100 µm gradient. These two samples, however, show a slight increase in electrode weight by 8 and 3%, respectively. The results reveal a strong correlation between surface temperature and mass loading, where steeper temperature gradients correspond to greater reductions in both temperature and mass.

This relationship can be applied during electrode manufacturing: a decrease in surface temperature signals a reduction in mass loaded onto the layer, while temperature variations across the surface indicate inconsistencies in mass loading. This highlights the potential for surface temperature analysis as an indirect indicator of mass loading, even detecting minor variations at low to middle mass loadings, making it a valuable tool for monitoring and ensuring consistent coating performance.

#### 3.2.2 Coatings with Fixed Wet‐Gap Heights

This chapter aims to investigate how fixed wet‐gap settings of the doctor blade influence the mass loading of the coating, the dry layer thickness, and the corresponding surface temperatures of a coating, showing the correlations between these parameters. The ultimate objective is to develop an estimation model for mass loading based on surface temperature data, showcasing the potential of thermal imaging as a real‐time quality control tool for detecting deviations in mass loading/thickness and for estimating mass loading in electrode fabrication. A fixed wet‐gap height maintains a constant distance between the doctor blade and the substrate, ensuring consistent wet film thickness across the coating width. As mentioned, this uniformity is essential for applications requiring stable properties, such as battery electrodes, solar cells, and other thin‐film technologies, where consistent thickness and material distribution are crucial for performance. Fixed wet‐gap settings enable better control over coating properties like layer thickness, mass loading, and drying behavior, directly impacting product quality.

Different coatings with varying mass loadings/thicknesses were achieved by setting the doctor blade to fixed wet‐gap heights of 20, 40, 60, 80, and 100 µm, resulting in a range of coating mass loadings/thicknesses. After following the drying protocol outlined in Section 2.3.1, thermal images of each coated layer were captured. For each wet‐gap height setting, eighteen electrodes were punched from each sample, allowing for a detailed comparison of surface temperatures with electrode mass loading and thickness through image registration. Thermal images of the coated layers, marked with visible symbols, were overlaid with scanned images of the punched coatings using the maximally MSER algorithm. This technique enabled accurate matching of surface temperatures to the positions of the punched electrodes.

**Figure 6:**
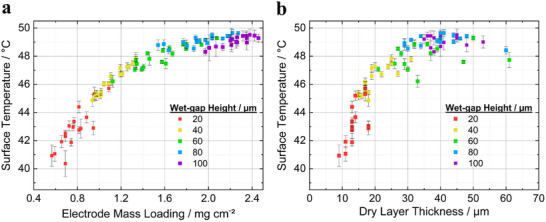
Dependence of the electrodes' average surface temperature on a) areal mass loading and b) dry layer thickness. Each data point represents mass loading or dry layer thickness of a 12 mm‐diameter circular electrode, with the standard deviation indicating temperature variation across the electrode surface. Eighteen data points (electrodes) were collected from the coating at each condition.

**Figure** **6**a shows the surface temperature distribution across coatings produced with fixed wet‐gap settings of 20, 40, 60, 80, and 100 µm. Despite the fixed settings, the final mass loading varied significantly. For instance, the 20 µm wet‐gap resulted in mass loadings ranging from 0.56 to 0.94 mg cm^−2^. Variability and standard deviation in mass loading decreased with higher wet‐gap settings. For coatings produced at 100 µm, most electrodes exhibited mass loadings between 2.2 and 2.4 mg cm^−2^, with a few as low as 2.0 mg cm^−2^. This variability in mass loading, even at fixed wet‐gap settings, can be attributed to the rheological properties of the nanoparticle slurry as mentioned before. Correspondingly, surface temperatures ranged from 40 °C for the low mass loadings to 49.5 °C for the high mass loading, further highlighting the relationship between mass loading and surface temperature. This increasing trend is in line with the data presented in Figure 5a, which further emphasizes the relationship between surface temperature and mass loading.

Detailed data analysis indicated a clear non‐linear relationship between mass loading and surface temperature. At lower mass loadings, the surface temperature increased significantly with additional material (increase in mass loading), as captured by the thermal imaging camera. However, as the mass loading continued to rise, the increase in surface temperature began to slow, resulting in a plateau around 2.0 mg cm^−^^2^. This suggests a saturation effect, where a further increase in mass loading results in diminishing returns on the finally measured temperature, i.e., sensitivity going down. This non‐linear behavior highlights how, beyond a certain point, additional material does not produce a proportional increase in IR radiation, likely due to material properties that limit the temperature increase at higher loadings and less likely represent limitations of the thermal camera.

Figure 6b plots the relationship between the dry layer thickness of the coated electrodes and their average surface temperature. The thermal imaging analysis revealed a clear correlation, with surface temperatures increasing as the dry layer thickness increased. For coatings with a thickness of around 10 µm, the average surface temperature was ≈40 °C, rising steadily to ≈49.5 °C as the thickness reached 50 µm. However, beyond a dry layer thickness of around 45 µm, a saturation effect was observed, where further increases in the dry layer thickness resulted in minimal changes in the surface temperature. Additionally, the spread of thickness values varied depending on the set wet‐gap, with the lowest standard deviation of ± 3 µm (average around 13 µm) for a 20 µm wet‐gap. The highest standard deviation of up to ≈ ±10 µm was recorded for coatings with a wet‐gap set equal to or above 60 µm. This trend demonstrates a significant relationship between coating thickness and temperature, suggesting that thermal imaging can serve as a reliable indicator for process control of the dry layer thickness.

Taken together, thermal imaging offers several strengths as a quality control tool in electrode fabrication. One of its key advantages is its ability to provide real‐time, non‐destructive measurements, allowing for continuous monitoring of coating parameters such as mass loading and dry layer thickness without interfering with the process. This makes it highly suitable for inline quality control, where rapid detection of deviations is essential. Additionally, the strong correlation between surface temperature and coating characteristics (such as mass loading and thickness) enables thermal imaging to serve as an effective indirect indicator of these properties, which can be challenging to measure directly in a production setting.

However, the results highlight some limitations of thermal imaging as well. While being effective for detecting larger variations in mass loading and thickness, its precision might reduce for small changes at high loadings or thicknesses due to saturation effects, where added material causes minimal temperature change. The observed saturation effect is not an inherent limitation of thermal imaging itself but rather a consequence of the material's heat transfer properties, such as thermal conductivity, specific heat capacity, and emissivity. The thickness at which saturation occurs is directly related to the ability of the material to conduct and store heat. In thicker Si/C coatings, heat is retained (heat capacity 0.7–0.9 J g^−1^·K^−1^) more effectively and distributed efficiently (2.7 W m^−1^·K^−1^ at 293 K),^[^ ^37^ ^]^ allowing the surface temperature to equilibrate with the heated bed temperature. In contrast, thinner Si/C coatings dissipate heat more rapidly, leading to lower surface temperatures in thermal imaging. Since these saturation limits are likely to vary across different materials, further research is required to define and characterize them for key materials used in R2R production lines. In this age of artificial intelligence (AI)‐driven advancements, developing a systematic database for battery and non‐battery materials will be essential. This database should include detectable mass loading and thickness thresholds in thermal imaging to optimize thermal imaging‐based quality control across broader manufacturing applications.

One possible approach to overcome the limitation caused by the saturation effect could be to apply a constant heat source, such as high‐energy light or IR radiation, to the top surface of the coating. This could modify the thermal gradient across the layer, reducing saturation while preserving thickness‐dependent thermal variations. However, this remains a hypothesis requiring further investigation and experimental validation, and its effectiveness is currently uncertain.

At this point, at least for Si/C being the material we target in this study, the maximum limit in mass loading for detecting a change in the thermal response was determined to be ≈2.4 g cm^−2^ (corresponding dry layer thickness averaging around 41 µm), which is much lower than the commercial graphite‐based anode where the mass loading is normally >10 mg cm^−2^ to balance a thick cathode for example, NMC 622 (specific capacity 180 mAh g^−1^).

This raises an important question: Is thermal imaging still a relevant tool for quality control at commercial scales for Si‐based anode materials? To address this, it is important to clarify a common misconception in the battery community that electrode mass loadings must always exceed 10 mg cm^−2^ for practical applications. This requirement primarily applies to graphite‐based or graphite/silicon composite anodes with low silicon content for automotive applications, where higher mass loadings are necessary due to the low specific capacity of graphite (≈372 mAh g^−1^). In contrast, pure silicon‐based anodes require significantly lower mass loadings because silicon has ≈10 times higher specific capacity (≈3500 mAh g^−1^).^[^ ^38^ ^]^ As a result, even at much lower mass loadings, silicon‐based anodes can achieve the same or higher areal capacity as conventional graphite‐based systems.

In this study, thermal imaging was able to detect a thermal response for Si/C‐based anode coatings up to ≈2.4 mg cm^−2^. This mass loading is in sufficient range to balance a commercial NMC 622 cathode with 20.2 mg cm^−2^ mass loading, 96% active material, and an areal capacity of 3.5 mAh cm^−2^ for automotive application.^[^ ^39^ ^]^ For an N/P ratio (ratio of expected areal capacity of anode to cathode) of 1.1, our calculations (Section S4.8, Supporting Information) show that only 1.34 mg cm^−2^ of silicon anode mass loading is required. That means even N/P ratios up to 2.0 can be covered (2.44 mg cm^−2^ Si anode). Therefore, thermal imaging as a quality control tool has a potential to be relevant and applicable for industrial‐scale Si/C‐based battery materials. However, for applications needing Si/C electrodes with mass loadings greater than the value of 2.4 mg cm^−2^, changes in thermal response may not be detectable, which could be a limitation of this testing method for Si/C anodes.

In summary, this analysis demonstrates the potential of thermal imaging as a non‐destructive tool with possible potential for real‐time quality control in electrode fabrication. By correlating surface temperature with both layer thickness and mass loading, thermal imaging enables reliable monitoring of the coating quality. Additionally, it provides a method for detecting defects, further enhancing quality control. Integrating this approach into production lines could significantly enhance electrode performance and improve quality control.

### 3.3 Mass Loading Prediction Using Thermal Imaging

#### 3.3.1 Model Development

This section investigates the potential to estimate mass loadings of Si/C composite electrodes using surface temperature data of the coating derived from thermal images. To explore the relationship between surface temperature and mass loading, we selected a combination of regression models and a machine learning approach. Piecewise, polynomial, and logarithmic regression models were selected for their simplicity and interpretability, making them well‐suited for capturing straightforward or segmented trends in the data.

Each regression model provides a different perspective on the temperature‐to‐mass‐loading relationship, from linear segments to polynomial and logarithmic patterns. Therefore, in addition to regression models, we employed the Random Forest method, a machine learning technique well‐suited for capturing complex, non‐linear interactions within a given dataset. Random Forest regression offers flexibility to model intricate patterns and detect subtle differences that traditional regression methods may overlook. We evaluated the accuracy of the models using R^2^, Absolute Error, MAE, and RMSE. In brief, R^2^ indicates the variance, the Absolute Error measures the raw difference between predicted and actual values, MAE measures the average error, and RMSE highlights larger errors. Together, these metrics offer a comprehensive view of the prediction reliability of each model and real‐world applicability. In such a way, this analysis helps to identify suitable predictive tools for monitoring mass loading in thin electrode coatings, potentially streamlining quality control and improving production efficiency.

**Figure 7:**
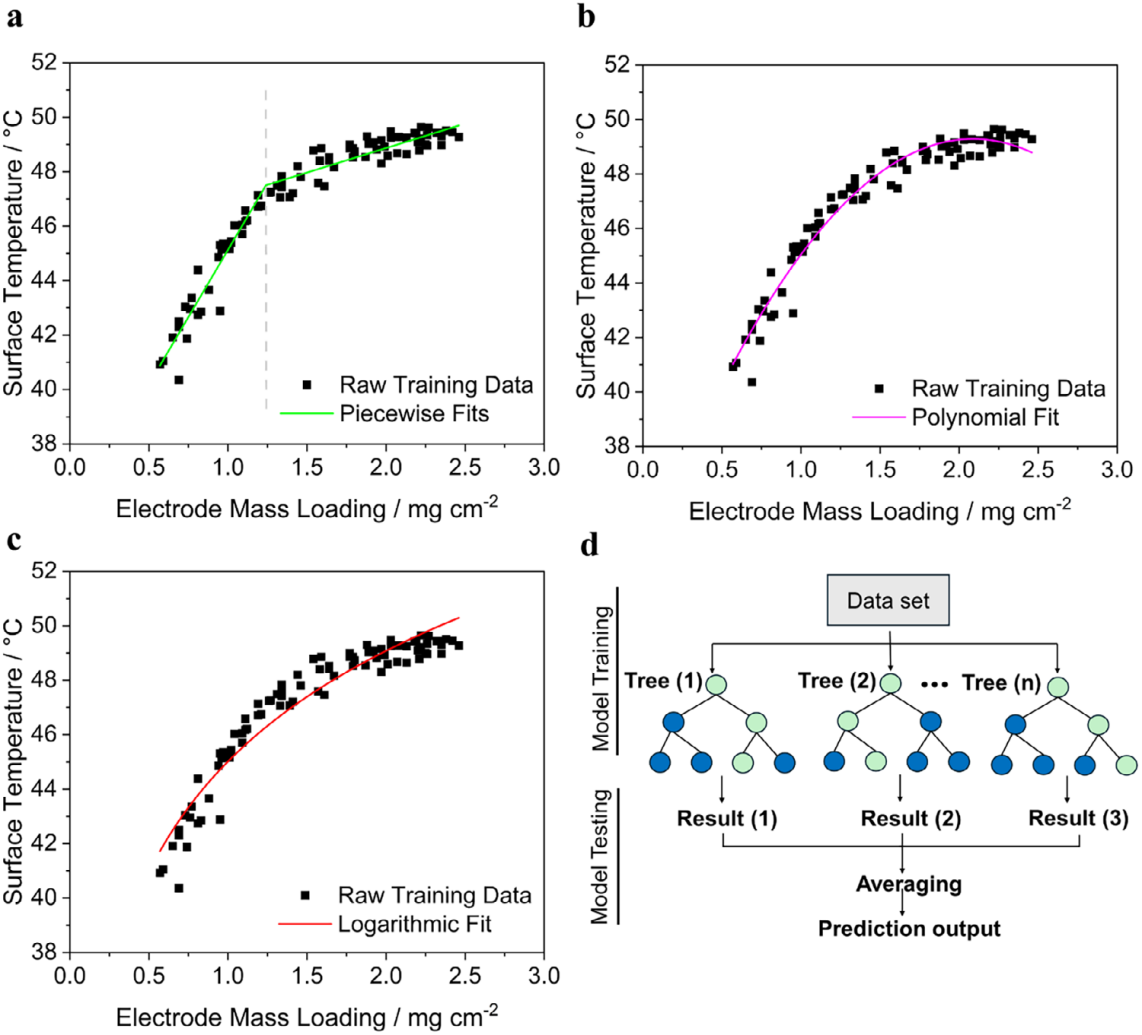
Model fits for electrode mass loading (mg cm^−2^) versus its average surface temperature (°C) using Origin software: a) Piecewise linear fit with a split at 1.23 mg cm^−2^; b) polynomial (degree 2) fit showing a peak trend; c) logarithmic fit capturing temperature response to mass loading; d) Random Forest process flow, highlighting ensemble prediction through multiple decision trees and result averaging. Note: mass loading and surface temperature data from 90 electrodes (18 electrodes per uniform coating condition) were used to establish fitting equations for conventional regression models and training the Random Forest model.

**Figure** **7** shows the fitted graphs of different regression models applied to the mass loading (0.5 to 2.5 mg cm^−2^) versus surface temperature data using Origin software, showing the relationship between mass loading and surface temperature.

Quantitative analysis using traditional regression models on this data yielded R^2^ values of 0.90 and 0.76 for the two segments of the piecewise fit, and 0.96 and 0.92, for the polynomial and logarithmic fits, respectively. For applying the machine learning tool Random Forest, the dataset was divided into a training set and a test set. The data was split into training and pre‐testing sets (80% for training and 20% for pre‐testing), and the model was trained using 100 decision trees, each built from random bootstrap samples of the data. The pre‐test set was then used to assess the model's pre‐performance, resulting in an R^2^ score of ≈0.94.

The fitted correlation equations for traditional models are shown as follows:

**Piecewise fit**

The fitted piecewise equation is as follows:

(4)

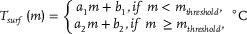




For this relationship:

*a*_1_ = 2.3 °C · *cm* ^2^ *mg* ^−1^, *b*_1_ = 15.7 °C; *a*_2_ = 1.1 °C · *cm* ^2^ *mg* ^−1^, *b*_2_ = 20.5 °C; and *m_threshold_* = 1.23 *g* *cm* ^−2^

**Polynomial fit**

This model, with a parabolic form, captures the non‐linear aspects of the data without segmenting it. The fitted equation is:

(5) $$T_{surf}={\left(-3.58m^{2}+14.98m+33.65\right)}^{\circ }C$$

**Logarithmic fit**

The logarithmic fit for the temperature versus mass loading data is given by:

(6) $$T_{surf}={\left(5.86\ln\left(m\right)+45.02\right)}^{\circ }C$$

With *T*_*surf*_(°C) representing the average surface temperature and *m* (mg cm^−2^) the mass loading of the electrode. The above equations (Equations 4–6) can be inverted to estimate the mass loadings of individual electrodes from the measured surface temperatures.

#### 3.3.2 Model Validation

Finally, we will validate the models and thereby compare them further with each other. To do so, new coatings were prepared at various wet‐gap settings, from which randomly 36 individual electrodes were cut out, and their surface temperatures were taken from thermal images using the MSER algorithm (see Section 2.4.1. Image processing). Mass loadings for each electrode were then estimated using three traditional regression models—piecewise, polynomial, and logarithmic fits—developed above. Additionally, after training the Random Forest regressor on training data (Figure 6a), these surface temperature measurements (from the new coating that was not part of the data shown so far) were input to predict mass loadings via the machine learning approach. The estimated mass loadings from each model were then compared to actual mass loadings obtained through direct measurement using a weighing technique.

**Figure 8:**
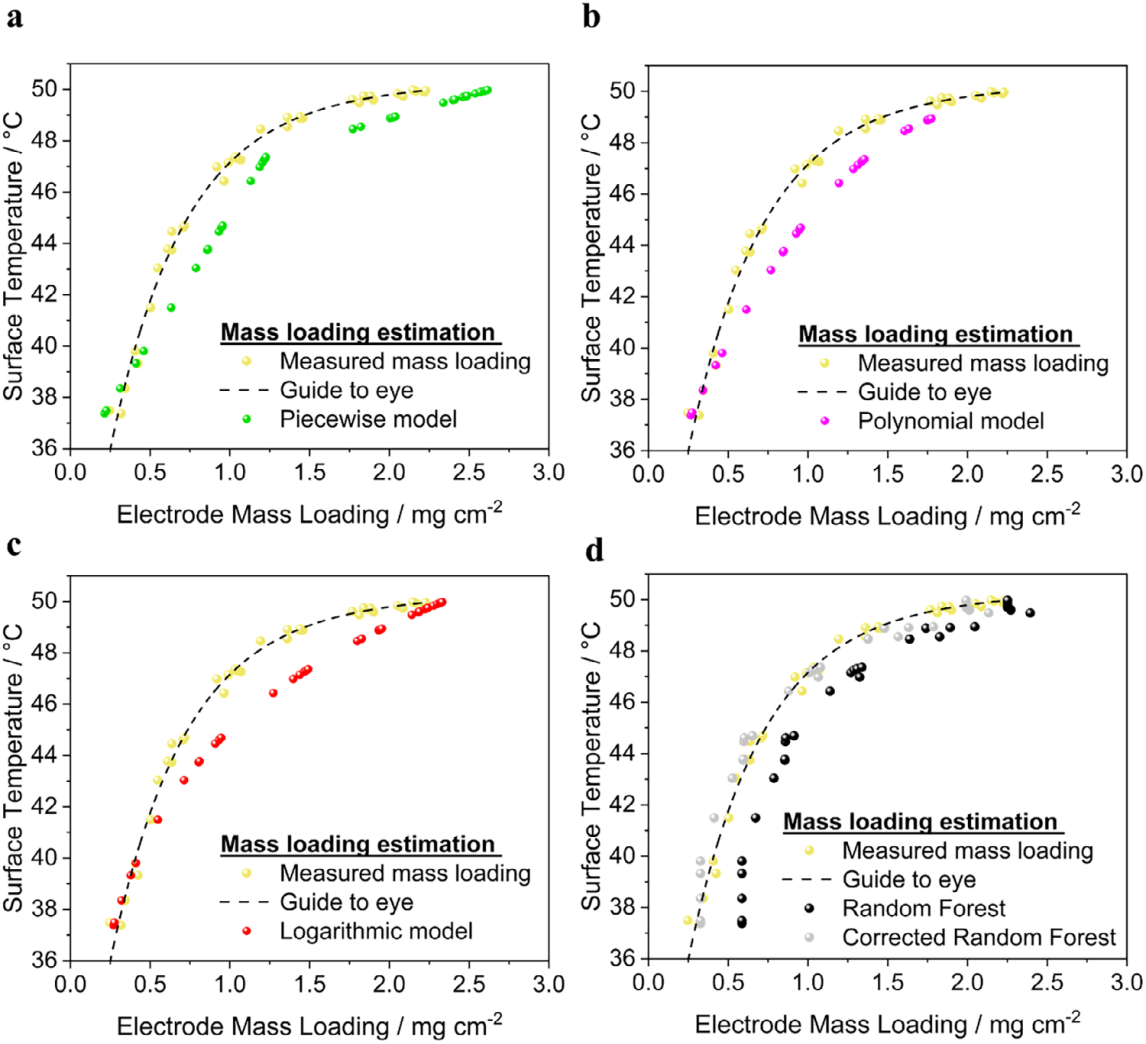
Comparison between actual mass loadings (measured by weighing) and estimated mass loadings derived from surface temperature measurements using four models: a) piecewise model, b) polynomial model, c) logarithmic model, and d) Random Forest method. Each subplot shows the model's performance in predicting the mass loading based on the surface temperature data. Note: 36 electrodes from a new coating were used to independently assess prediction performance.

**Figure** **8** presents a comparison between the actual mass loadings, measured by weighing, and the estimated mass loadings derived from surface temperature measurements using the three traditional regression models (piecewise, polynomial, and logarithmic fits) and the machine learning‐based Random Forest regressor.

Figure 8a shows the estimated results of the mass loadings from thermal data using the piecewise linear model, with a comparison to the measured values. The results show that the piecewise linear model divides the estimated mass loadings data into two linear segments, estimated using one equation below a cut‐off temperature of 47.3 °C (corresponding to a cut‐off mass loading of 1.23 mg cm^−2^) and another above it. This approach effectively estimates lower mass loadings using thermal data but struggles to capture the non‐linear relationship, particularly at the curvature and for higher mass loadings in the mass loading versus surface temperature correlation.

Figure 8b presents mass loading predictions using the polynomial model, comparing them with the measured values. The polynomial model shows an overall performance similar to the piecewise fit: it closely estimates lower mass loadings but struggles to capture the curved relationship at higher mass loadings. An additional limitation of the polynomial model is that at higher surface temperatures, the predictions for thicker electrodes became problematic due to the non‐real solutions. This occurs because the polynomial model is solved using a quadratic equation, where imaginary solutions can arise under certain conditions. Therefore, this model does not even cover a full range of electrode mass loadings.

In Figure 8c, the mass loading predictions based on surface temperatures using the logarithmic model are shown, with a comparison to the measured values. While logarithmic models are often effective for data trends that stabilize at high values, this model also predicted the mass loadings at extreme ends well. However, with regard to curvature, the prediction is very poor.

Finally, Figure 8d shows mass loading predictions for electrodes, derived from surface temperature data using the Random Forest regressor, compared with the measured values. This machine learning model offers flexible estimations of the mass loading‐temperature relationship, accurately modeling the curvature (dark spheres in Figure 8d). Initially, the machine learning model had difficulty in estimating very low mass loadings (lower surface temperatures) due to the absence of surface temperature data for mass loadings below 0.5 mg cm⁻^2^ in the training data set. This is in line with the expectation as machine learning models are known to get ineffective when they go out of the trained parameter space. Therefore, we added temperature data (six electrodes) for mass loadings below 0.5 mg cm^−2^. It significantly improved the model's performance (Figure S9, Supporting Information) for estimating low mass loadings, highlighting its adaptability. Unlike conventional models, which require a new equation with each update, the Random Forest regressor can incorporate new data whenever they become available to continuously improve its predictions.

Furthermore, the Random Forest regressor outperformed conventional models by better capturing the trend in the data. In an industrial setting, process parameters are typically controlled to maintain consistent product quality. Since the Random Forest regressor's mass loading predictions exhibited homogeneous deviations, it allows for the application of correction factors to adjust the model's output, offering greater control over the process. Therefore, a constant correction factor of 0.26 mg cm^−2^ (which is the knowledge of error we obtained from pre‐testing in the model development where R^2^ was 0.94) was applied to the data. As can be seen in grey data points, an excellent match of estimated and measured mass loadings was observed. Application of such correction factors is not possible for conventional models because they do not follow the exact trend in their estimations.

#### 3.3.3 Error Analysis in Mass Loading Predictions

**Figure 9:**
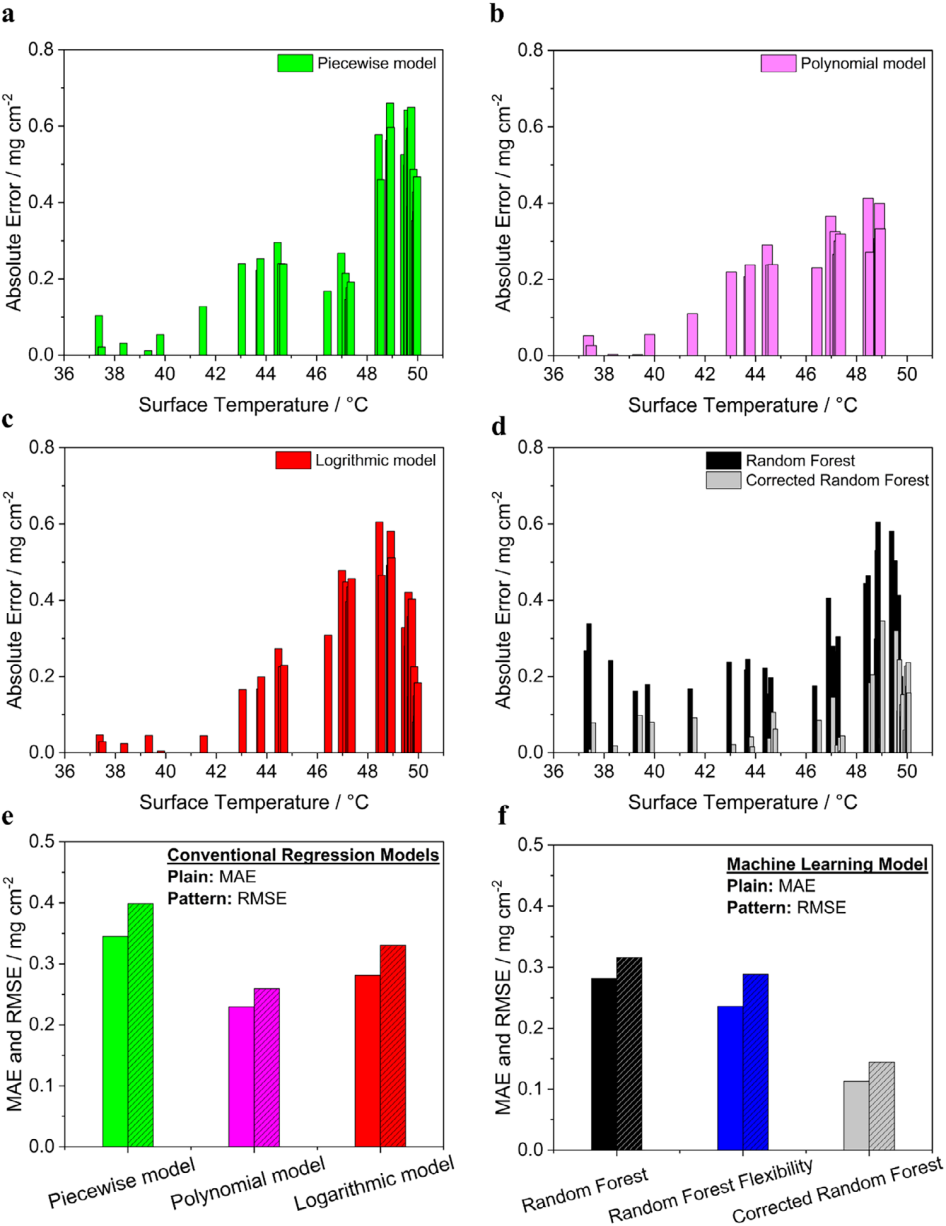
Comparison of absolute errors in the predicted mass loading across different regression and machine learning models as a function of electrode average surface temperature. a) Piecewise (green), b) polynomial (pink), c) logarithmic (red), and d) Random Forest (dark) and corrected Random Forest (grey) models. The bottom row presents the MAE and RMSE for each model category, with results of the conventional regression models on the left and of the machine learning‐based model on the right.

**Figure** **9** provides a comparative analysis of the absolute errors in mass loading predictions across different modeling approaches—conventional regression, Random Forest (trained at *m*_*mass* *loadings*_ = 0.5‐2.5 mg cm^−2^), and corrected Random Forest—as a function of the electrode average surface temperature. Additionally, the MAE and RMSE for each model, including Random Forest flexible (trained at *m*_*mass* *loadings*_ = 0.25–2.5 mg cm^−2^) as a function of the electrode average surface temperature, along with the MAE and RMSE for each model.

Figure 9a–d shows a comparative analysis of absolute errors in mass loading predictions across different models. The piecewise model performs well at low mass loadings (≤0.46 mg cm⁻^2^) but shows increasing error, up to 0.65 mg cm⁻^2^, at higher mass loadings due to a poor fit (R^2^ = 0.76) in the second linear segment. The polynomial model accurately predicts lower mass loadings but becomes inconsistent at higher mass loadings and fails due to non‐real solutions, limiting its applicability. The logarithmic model poorly aligns with the data, as it does not capture a plateau at higher temperatures, leading to discrepancies in predictions above 42 °C. In contrast, the Random Forest regressor shows relatively lower absolute error at increasing mass loadings, as it better follows the curvature trend. Applying the correction factor further reduces the maximum MAE from 0.60 to 0.35 mg cm^−2^ for estimation of the highest mass loading.

Figure 9e,f shows the MAE and RMSE values for various models used to predict the mass loading from the surface temperature of the electrode. Among the conventional regression models, the piecewise model exhibited the highest deviations, with an MAE of 0.34 and an RMSE of 0.39, indicating its limited accuracy. The polynomial model showed improved performance, achieving an MAE of 0.23 and an RMSE of 0.25. The polynomial model provided a continuous equation for mass loading predictions and performed slightly better in predicting lower to mid‐range mass loadings than the piecewise fit, but overall, it was less reliable due to its non‐flexibility and inability to give any prediction about higher mass loadings. The logarithmic approach displayed moderate error levels, with an MAE of 0.29 and an RMSE of 0.33, placing it between the piecewise and polynomial models in accuracy. Again, the Random Forest regressor demonstrated the lowest errors among the conventional regression models, with an MAE of 0.28 and an RMSE of 0.31, which were further reduced to 0.23 and 0.28, respectively, when temperature data in the very lower mass loading range (flexible Random Forest) was also included in the training of the model. Also as already seen above, the accuracy of the model improved significantly upon applying a correction factor, resulting in an MAE of 0.11 and an RMSE of 0.14 (corrected Random Forest), the lowest error rates among all models evaluated in this study, highlighting its superior predictive capability.

In summary, the comparative analysis summarized in Figure 9 highlights the strengths and limitations of both conventional regression and machine learning models in predicting mass loading based on electrode surface temperature. While conventional regression models such as piecewise, polynomial, and logarithmic fits showed varying levels of accuracy, each had limitations, particularly at higher mass loadings or temperature ranges and their non‐flexibility. The Random Forest regressor outperformed these traditional approaches, with further improvements that could be achieved through correction factors that significantly reduced prediction errors. However, it is important to note that introducing correction factors adds an additional parameter to the model, which requires careful consideration and calibration for each processing material.

A potential limitation arises when the materials used in production undergo even slight changes, as model predictions are typically based on data from previously used materials. This challenge is particularly relevant in battery manufacturing, where small variations in formulation properties are common due to differences in the materials provided. Such changes could reduce the model's prediction accuracy unless it is updated with new data. This highlights the importance of using flexible machine learning models like Random Forest, which can effectively incorporate new datasets to adjust predictions to evolving material formulations or properties. By enabling continuous adaptation, these models ensure robust performance in dynamic environments where material variability is unavoidable. In this study, the corrected Random Forest model demonstrated the best performance, delivering the lowest MAE and RMSE values and confirming its effectiveness for accurate mass loading estimation across a wide range of temperatures.

### 3.4 Investigation of Time‐Dependent Drying Effects

Investigating drying curves is essential for optimizing both the drying process and the web speed in R2R electrode manufacturing. The drying process significantly affects key electrode properties, including adhesion, rate capability, and capacity. Fast drying rates can cause defects like binder migration, leading to poor adhesion and uneven binder distribution.^[^ ^40^ ^]^ In an R2R process, it is essential to carefully balance the coating speed with the drying rate. Typically, excessively fast line speeds can lead to incomplete drying and introduce defects, while slower speeds may enhance drying but reduce the overall production efficiency. Additionally, drying that is too slow or takes place at lower temperatures can destabilize the film, causing issues like de‐wetting, further compromising the quality of the coating.^[^ ^41^ ^]^ Therefore, understanding the drying behavior is crucial for achieving optimal coating quality and efficient production.

In this experiment, the thermal camera was positioned ≈120 mm above the heated bed to capture accurate surface temperature measurements. Prior to slurry casting, the heated bed was pre‐set to the target temperatures of 30, 60, and 90 °C. Coatings were applied with gap heights of 50 and 100 µm, and a fixed width of 60 mm. Surface temperatures of the coatings were recorded continuously over a period of 10 min. For the analysis, the recorded temperatures were averaged to assess the overall surface temperature profile of each coating condition. The shrinkage of electrode coatings during drying, as shown in Figure S10 (Supporting Information), was evaluated by comparing the wet‐gap height with the final dry thickness. Coatings with a 100 µm wet‐gap exhibited the highest shrinkage (60%), while those with a 20 µm gap shrunk the lowest (31%). This variation is likely due to differences in solvent evaporation rates in such a way that coatings with higher wet‐gap settings retain more solvent initially, leading to greater shrinkage during drying.

**Figure 10:**
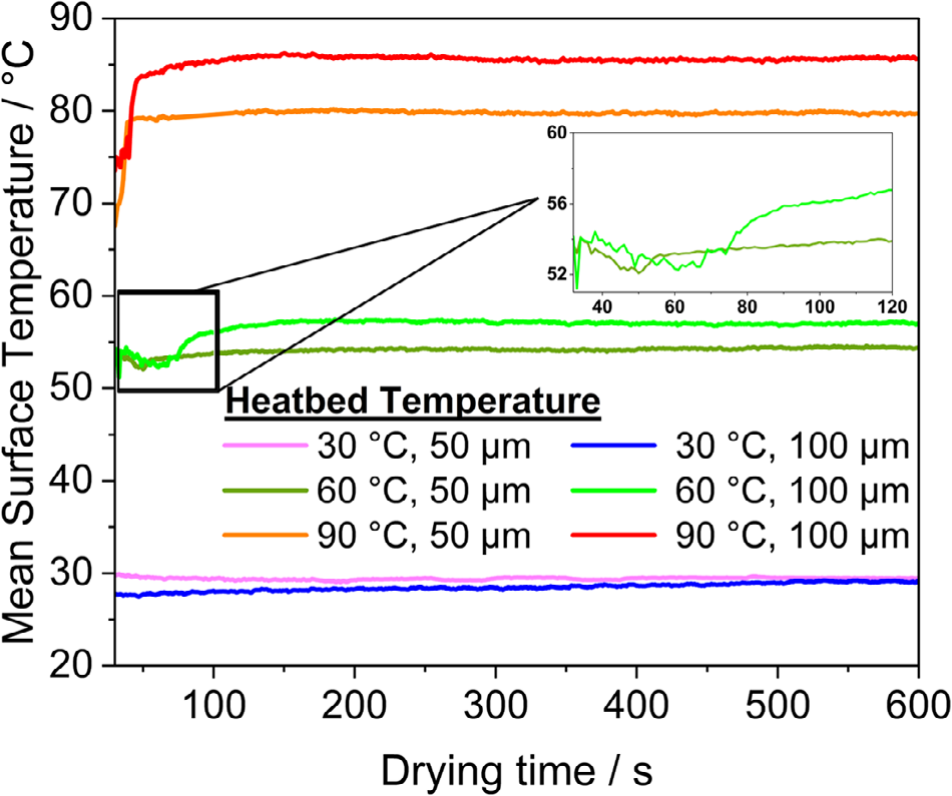
Evolution of mean surface temperature over time for coatings generated from varying initial wet film thicknesses (100 and 50 µm) at different heated bed temperatures (90, 60, and 30 °C). The inset graph shows the fall and rise phases of the samples coated at a heated bed temperature of 60 °C. Note: The lowest temperature threshold values used to generate the drying curves were manually set in the MATLAB code based on the apparent thermal image temperature of the copper substrate (low emissivity), allowing selective tracking of the higher‐emissivity coating. Thresholds were set to 25, 30, and 40 °C for heated bed temperatures of 30, 60, and 90 °C, respectively.

**Figure** **10** shows the evolution of mean surface temperature over time for coatings with different initial wet film thicknesses at various drying temperatures, controlled by the heat‐bed temperature. The camera was started before the coating was applied to the copper surface. The displayed temperatures are shown from the moment the applicator has fully coated the layer. This setup allows real‐time observation of the drying behavior of coated electrodes, highlighting how solvent evaporates and how surface temperature changes during the drying process.

The evolution of surface temperatures during drying is influenced by both the heated bed temperature and the wet‐gap setting of the doctor blade. At a heated bed temperature of 90 °C, the surface temperature of the coating prepared with a wet‐gap of 100 µm (resulting in a dry layer thickness of 40 µm) stabilizes at 85 °C, while the coating with a wet‐gap of 50 µm (dry layer thickness of 20 µm) stabilizes at 80 °C within 150 s. At 60 °C, both coatings initially exhibit a temperature drop of ≈2 °C within the first 15 s due to transient evaporation, followed by a gradual rise. The coating generated with a 50 µm wet‐gap stabilizes at 54 °C after 10 s, whereas the coating generated with a 100 µm wet‐gap stabilizes at 57 °C, requiring ≈40 s to do so. At 30 °C, drying occurs predominantly through natural convection near room temperature, with minimal surface temperature variation.

The observations discussed above can also be visualized in a thermal video. For instance, Video S1 (Supporting Information) showcases sample coatings prepared with different wet‐gap heights (50 and 100 µm) using a doctor blade, with copper foil placed on a heated bed set to 60 °C. In Video S1a (Supporting Information), for a wet‐gap height of 50 µm, drying occurred almost instantaneously compared to the coating prepared at a 100 µm wet‐gap height (Video S1b, Supporting Information). In both samples, the reduction in surface temperature correlated with a visible drying front at the beginning of the process. As drying progressed, the solvent diminished into smaller islands, as seen in the thermal video. Once these islands disappeared, the surface temperature increased, indicating that the observed reduction in surface temperature was likely caused by the heat required for evaporation. However, the drying rates and overall drying times were noticeably different, with the 50 µm sample drying faster than the 100 µm sample. Moreover, in thermal imaging video recordings of the drying process at a 100 µm wet‐gap and 60 °C (Video S1b, Supporting Information), pinhole‐type defects became visible during the drying phase. Additionally, when a slurry drop was deposited on the coating surface before the main coating process, it resulted in thicker regions in the final electrode (Figure S11, Supporting Information). These observations suggest that thermal imaging can capture defect formation dynamics during drying. However, a detailed investigation of defect evolution mechanisms would require further studies.

The above observations reveal how the wet‐gap setting influences the drying process. Coatings prepared with larger wet‐gap settings retain more solvent, requiring longer drying times due to the increased solvent content and slower diffusion of solvent to the surface. In contrast, coatings prepared with smaller wet gaps dry faster because they contain less solvent, allowing for quicker diffusion and more efficient heat transfer. These differences are particularly pronounced at intermediate temperatures (60 °C), where transient evaporation and stabilization times differ significantly with coating thickness.

Overall, the heated bed temperature is the dominant factor in determining the drying speed. At higher temperatures (90 °C), drying progression stabilizes rapidly. At 60 °C, an intermediate stabilization occurs after an initial cooling phase, while at 30 °C, drying is slow and primarily driven by convection. However, the wet‐gap height setting also plays a significant role, as coatings with higher wet‐gaps require more time to dry due to prolonged evaporation processes and exhibit higher final surface temperatures.

## 4 Conclusion

This study successfully demonstrated the potential of thermal imaging as a non‐destructive quality control tool for detecting coating defects and analyzing mass loading and thickness variations in the production of silicon‐based thin coatings. Thermal imaging effectively identified critical defects such as streaks, pinholes, and chatter marks, with streaks reducing the surface temperature by up to 15 °C and chatter marks showing periodic variations of ≈5 °C. These results highlight the method's exceptional sensitivity, surpassing traditional visual and laser‐based inspection techniques in detecting subtle and performance‐critical flaws.

Thermal imaging also revealed clear correlations between surface temperature, mass loading, and coating thickness. For instance, a 100 µm wet‐gap coating exhibited a surface temperature of 49.5 °C and a mass loading of 2.4 mg cm^−2^, whereas a 20 µm wet‐gap resulted in lower temperatures around 40 °C, corresponding to a mass loading of 0.6 mg cm^−2^. Furthermore, integrating machine learning, particularly Random Forest models, enhanced the predictive capabilities of thermal imaging, offering higher accuracy in estimating mass loading compared to traditional regression approaches. These findings open up the possibility that thermal imaging could be explored as a tool for real‐time monitoring and optimization in R2R processes. While our study was conducted in a stationary setup, the strong correlation between surface temperature and coating thickness/mass loading, along with the method's speed and non‐destructive nature, points to its potential for future adaptation. However, further validation in a dynamic setting is needed.

Finally, the study examined drying dynamics and their dependence on heatedbed temperature and wet‐gap height. Coatings dried at 90 °C stabilized within 150 s, while drying at lower temperatures was slower and characterized by more gradual temperature increases. Drying dynamics also influenced the final electrode thickness, with coatings from a 100 µm wet‐gap experiencing up to 60% shrinkage during drying. These observations emphasize the importance of controlling both heated bed temperature and wet‐gap height to achieve desired electrode properties.

In conclusion, the integration of thermal imaging into industrial‐scale R2R production offers a promising pathway for real‐time monitoring and correction of defects, mass loading, and thickness variations. This approach would enhance quality control, improve the mechanical and functional performance of coatings, and contribute to more reliable and efficient production processes. Moreover, it is also capable of providing kinetic inline or at‐line data that could potentially be applied in future studies for validating numerical drying models.

## Conflict of Interest

The authors declare no conflict of interest.

## Supporting information



Supporting Information

Supplemental Video 1

Supplemental Video 2

## Data Availability

The relevant data for this study is accessible on Zenodo at https://doi.org/10.5281/zenodo.15172703
