# Supplementary material for: Thermal Imaging for Quality Control in Thin Silicon‐Based Coatings for Lithium‐Ion Batteries: Defect Detection, Drying Dynamics, and Machine Learning‐Based Mass Loading Estimation
Source: Small Methods. 2025 Apr 14;9(7):2402079. doi: 10.1002/smtd.202402079 (PMC12285618; doi:10.1002/smtd.202402079)
Supplement: Supplementary file 1 — Supporting Information [file SMTD-9-2402079-s003.docx]

*Supporting Information (SI)*

Thermal Imaging for Quality Control in Thin Silicon-Based Coatings for Lithium-Ion Batteries: Defect Detection, Drying Dynamics, and Machine Learning-Based Mass Loading Estimation

*Adil Amin,^†^ Philipp Valentin Geiping,^†^ Ahammed Suhail Odungat, Fatih Özcan, Doris Segets^*^*

A. Amin, P. V. Geiping, A. S. Odungat, Dr. F. Özcan, Prof. Dr.-Ing. habil. D. Segets

Institute for Energy and Materials Processes – Particle Science and Technology (EMPI-PST), Carl-Benz-Straße 199, 47057, Duisburg, Germany

*E-mail: [doris.segets@uni-due.de](mailto:doris.segets@uni-due.de)

Dr. F. Özcan, Prof. Dr.-Ing. habil. D. Segets

Center for Nanointegration Duisburg-Essen (CENIDE), University of Duisburg-Essen (UDE), Carl-Benz-Straße 199, 47057, Duisburg, Germany

†These authors contributed equally to this manuscript

# 1. Materials


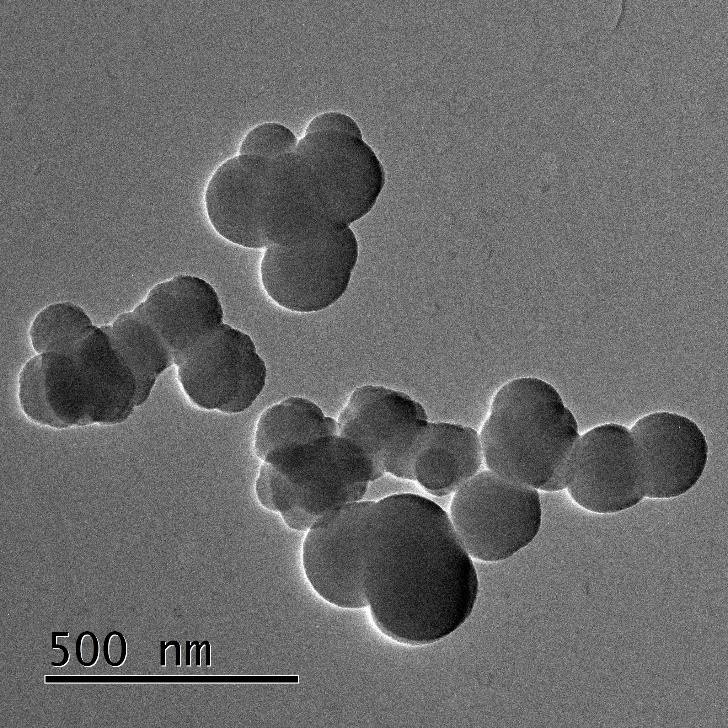


Figure S1. Transmission electron microscopy (TEM) image of Si/C nanoparticles synthesized through a gas-phase process in a hot-wall reactor.

# 2. Experimental

## 2.1. Slurry making procedure

For making the battery slurry a two-step procedure was followed. In the first step, Poly (acrylic) acid, Carbon black (C-65), and half of the deionized water were pre-mixed in a ball mixer (IKA tube mixer) at 4000 rpm for 3 minutes with 5 mm zirconium oxide milling balls (3 balls). After this initial dispersion, the active material and remaining water were added, followed by an additional mixing step under the same conditions to achieve a homogeneous slurry.


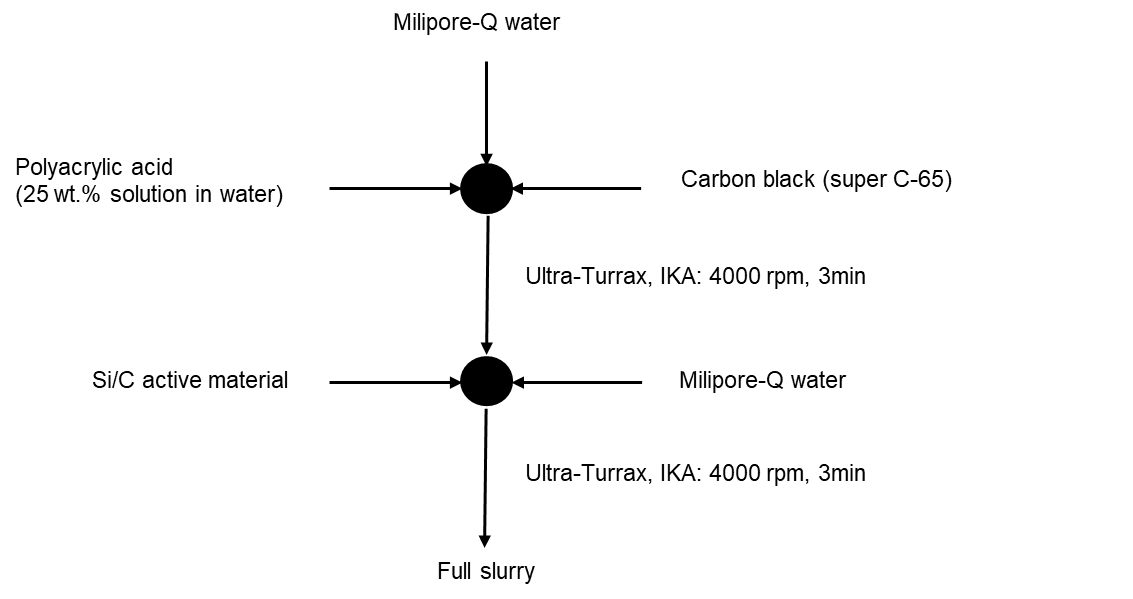


Figure S2. Two-stage strategy for the preparation of anode slurries. Reproduced under terms of the CC-BY license.^[1]^ © 2023, Adil Amin, Moritz Loewenich, Stefan O. Kilian, Theresa Wassmer, Stefan Bade, Julia Lyubina, Hartmut Wiggers, Fatih Özcan, and Doris Segets. Published on behalf of The Electrochemical Society by IOP Publishing Limited.

## 2.2. Coating defect types

Table S1. Typical defect types in roll-2-roll manufacturing. Selected defect types and their shapes are clustered from various sources mentioned in reference 2. ^[2]^

| Defect type | Description | Cause for occurrence |
| --- | --- | --- |
| 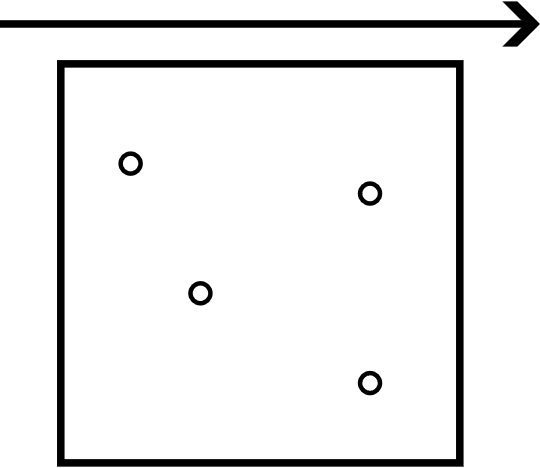Pinholes | circular regions depleted of coating | air bubbles in the suspension, gas releasing during coating and drying |
| 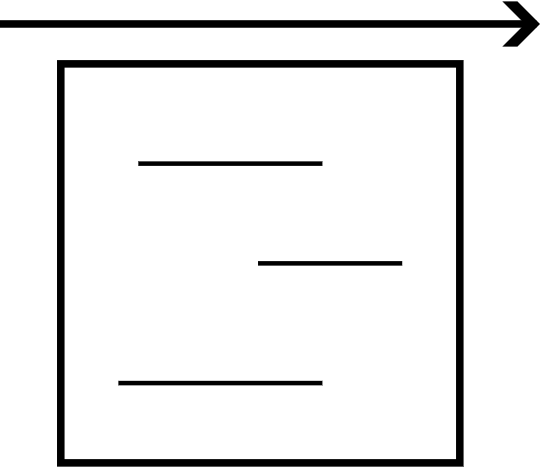Streaks | elongated lines parallel to the direction of coating | obstruction of the blade gap, high slurry viscosity, inhomogeneous mixing of slurry components |
| 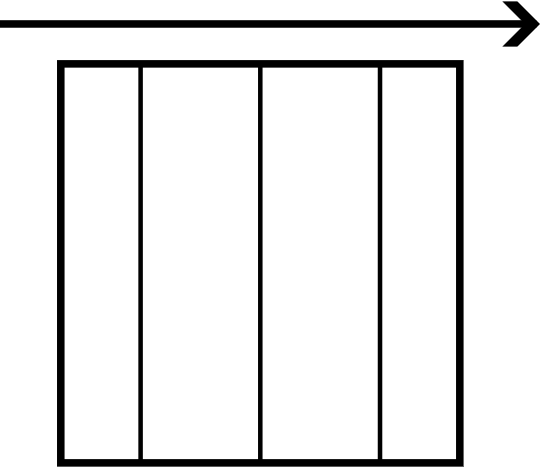Chatter | lines across the coating perpendicular to the coating direction | mechanical vibrations of the blade or other machine parts |

# 3. Theoretical Basis

The Random Forest approach is based on an ensemble learning method, which combines the predictions of multiple individual models (decision trees) to improve accuracy and robustness. Specifically, a Random Forest consists of a collection of decision trees, where each tree is trained on a randomly selected subset of the data and features. The final prediction is made by aggregating the outputs of all the trees, typically through averaging for regression or majority voting for classification tasks.

## 3.1. Key components of a Random Forest ^[3]^

- Decision Trees:

A decision tree is a flowchart-like structure used to make decisions. It splits the data into branches based on feature values to make predictions. Each node in the tree corresponds to a decision based on a feature, and each leaf node provides the predicted output.

- Bootstrap Aggregating (Bagging):

Random Forest uses a technique called bagging (Bootstrap Aggregating). It creates multiple decision trees by taking random subsets of the training data.

- Random Feature Selection:

When building each decision tree, Random Forest selects a random subset of features (instead of using all features) at each split in the tree. This helps to make the trees more diverse, which improves the performance of the ensemble. Also, the random feature selection helps to reduce correlations between trees, ensuring that the Random Forest does not rely too heavily on a few features and enables better generalization.

## 3.2. Step for building Random Forest method:

1. Random Data Sampling:

The Random Forest algorithm generates multiple bootstrap samples (random subsets of the training data). Each sample is used to train one decision tree.

1. Training Multiple Decision Trees:

For each bootstrap sample, a decision tree is trained, but during the training, only a random subset of features is considered for each decision split. This is what makes the model "random."

1. Making Predictions:

After all trees are trained, the Random Forest makes predictions: For regression (continuous output), the predicted value is the average of the predictions from all the trees.

1. Output Aggregation:

The final prediction is aggregated across all the decision trees. By averaging predictions (for regression), the Random Forest reduces the likelihood of errors from any individual tree, leading to better performance.

# 4. Supporting results

## 4.1. Generation of chatter defects


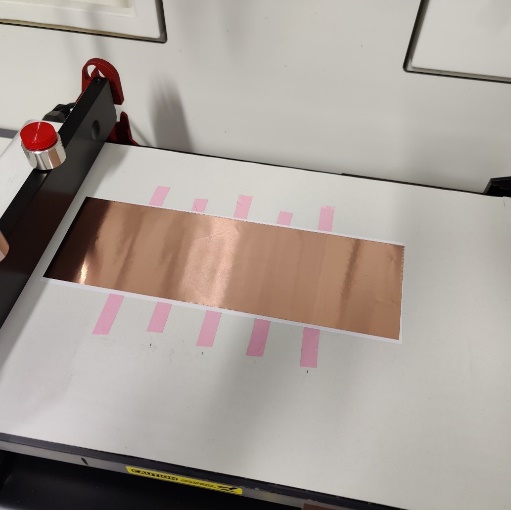


Figure S3. Generation of chatter defects by applying paper tapes at the bottom of the copper sheet to generate ripples at certain positions.

## 4.2. Filter-out streaks from thermal image


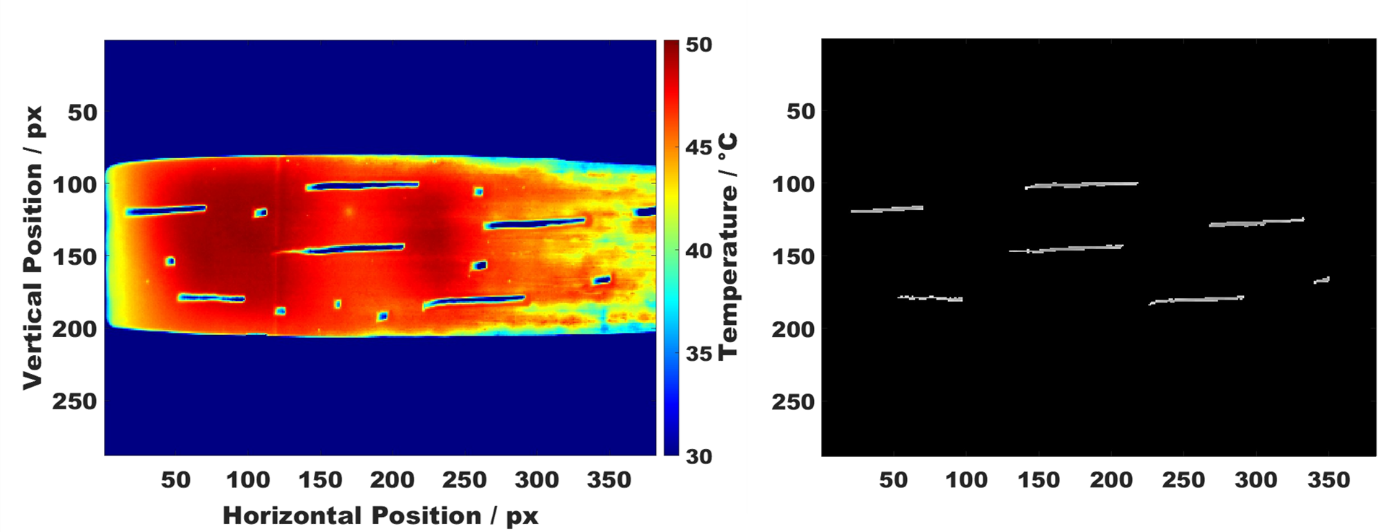


Figure S4. a) Thermal image of the coating displaying both streak and non-streak defects. b) Streak defects isolated using a dimensionality filter, effectively distinguishing them from other defect types.

##
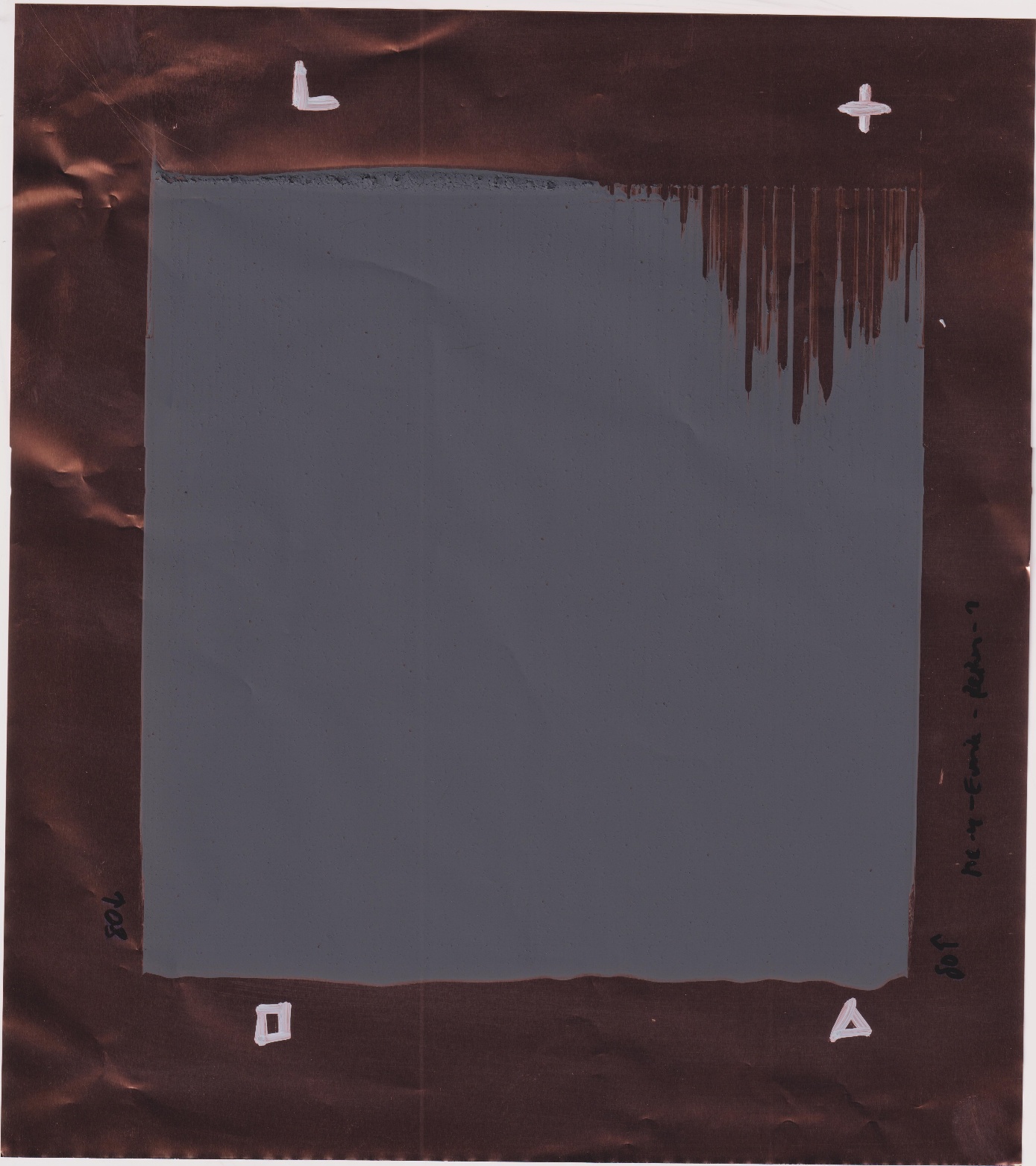
4.3. Photo scan of full coating with pinhole defects

Figure S5. Photo scan of full test coating showing pinhole defects.

## 4.4. Filter-out chatter defects from thermal image


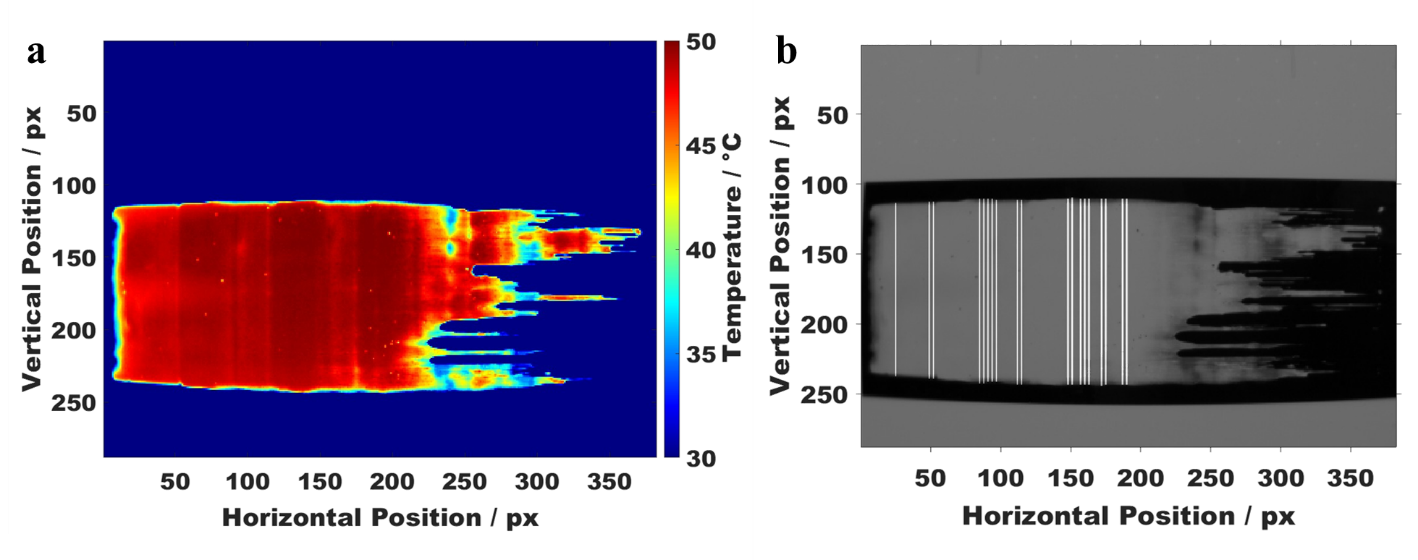


Figure S6. a) Thermal image of the coating displaying chatters and their temperature. b) Chatter defects isolated using a Sobel filter and hit-and-miss operation.

## 4.5. Filter-out pinhole defects from thermal image


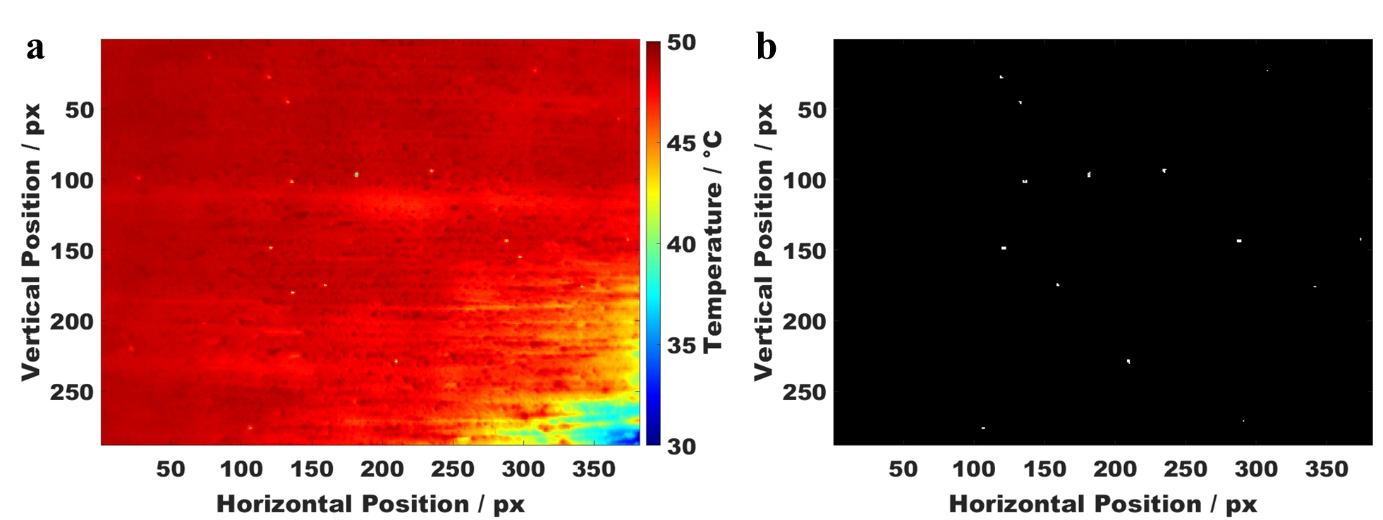


Figure S7. a) Thermal image of the coating displaying pinhole defects and their temperature. b) Pinhole defects isolated using a dimensionality filter.

## 4.6. Defects outside field of view


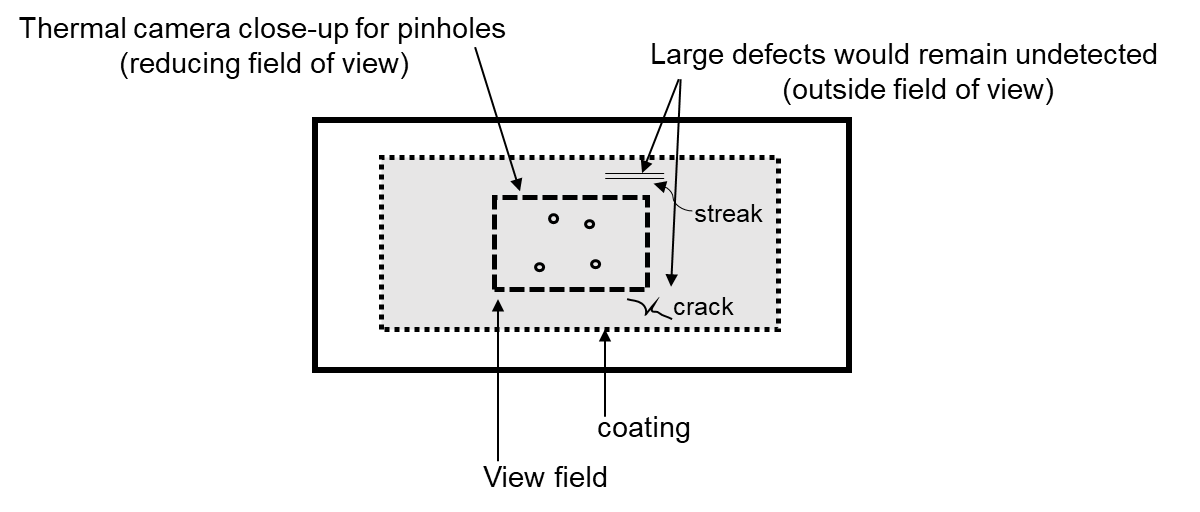


Figure S8. Schematic showing the trade-off between spatial resolution and field of view in thermal imaging. Moving the thermal camera closer to the electrode surface enhances resolution for detecting small-scale defects (e.g., pinholes, agglomerates), but narrows the field of view, potentially leaving out defects located outside the captured area. This is a conceptual illustration and not an actual thermal image.

## 4.7. Mass loading and dry layer thickness

The mass loading (mg cm^-2^) of the electrodes is defined as the mass of solid material coated per unit area of electrodes. Electrodes with a diameter of 12 mm were punched from the copper sheets using an electrode cutting plier *EL-Cut* (EL-Cell GmbH, Germany). The weight of the electrodes was measured on a high-precision laboratory scale. The mass of the loaded slurry was determined by subtracting the average mass of the 12 mm diameter bare copper foils ($\cong$ 18.13 ± 0.05 mg) from the average mass of the coated electrodes of each category. The areal mass loading of electrodes was determined using Equation (SI 1) as follows:

$$\text{Areal mass loading = Mass of coated solid material (mg)}\text{ }\text{/}\text{ }\text{Area of electrode (}\mathrm{cm}^{2}\text{)}\text{ (}\text{SI }\text{1)}$$

The coating heights/thickness of the dried electrodes were measured using a Digital-Mikrometer (Pollin Electronic Ladengeschäft). The displayed resolution was 1 μm. To determine the height/thickness of the coating, the thickness of the copper foil, recorded at approximately 18 μm, was subtracted.

## 4.8. Calculations for required mass loading for Si-rich anodes

For automotive applications, the typical cathode areal capacity ranges between 3–4 mAh cm^-2^. The mass loading of a commercial NMC-based cathode (including NMC, binder, and carbon) is approximately 20.2 mg cm^-2^ based on the specific capacity of NMC622 (180 mAh g^-1^) and an active material fraction of 0.96.

To calculate the required mass loading of a silicon anode (specific capacity 3579 mAh g^-1^) needed to balance the cathode, we assume a cathode areal capacity of 3.5 m Ah cm^-2^. In Si-rich anodes, the active mass fraction of silicon is typically 0.80. To ensure proper electrode balancing, we consider an N/P ratio of 1.1.

The N/P ratio is defined as:

$$\frac{N}{P}= \frac{Areal capacity of anode}{Areal capacity of cathode} Equation S1$$

Rearranging for the areal capacity of the anode:

Areal capacity of anode = $\frac{N}{P}\times Areal capacity of cathode$ = 1.1 × 3.5 = 3.85 mAh cm^-2^

The mass loading of the silicon anode is calculated as:

$$Mass loading= \frac{Areal capacity of anode}{Specific capacity of silicon\times Active material fraction in anode} Equation S2$$

$$Mass loading=1.34 mg {cm}^{-2}$$

## 4.9. Random Forest flexibility (0.25-2.5 mg cm^-2^)

**

**

Figure S9. Improved performance of the Random Forest regressor in estimating unknown low mass loadings after incorporating temperature data from lower mass loading ranges into the training dataset. This demonstrates the model’s flexibility and its ability to adapt and improve as new data becomes available.

## 4.10. Coating Shrinkage at various wet-gap settings of doctor blade


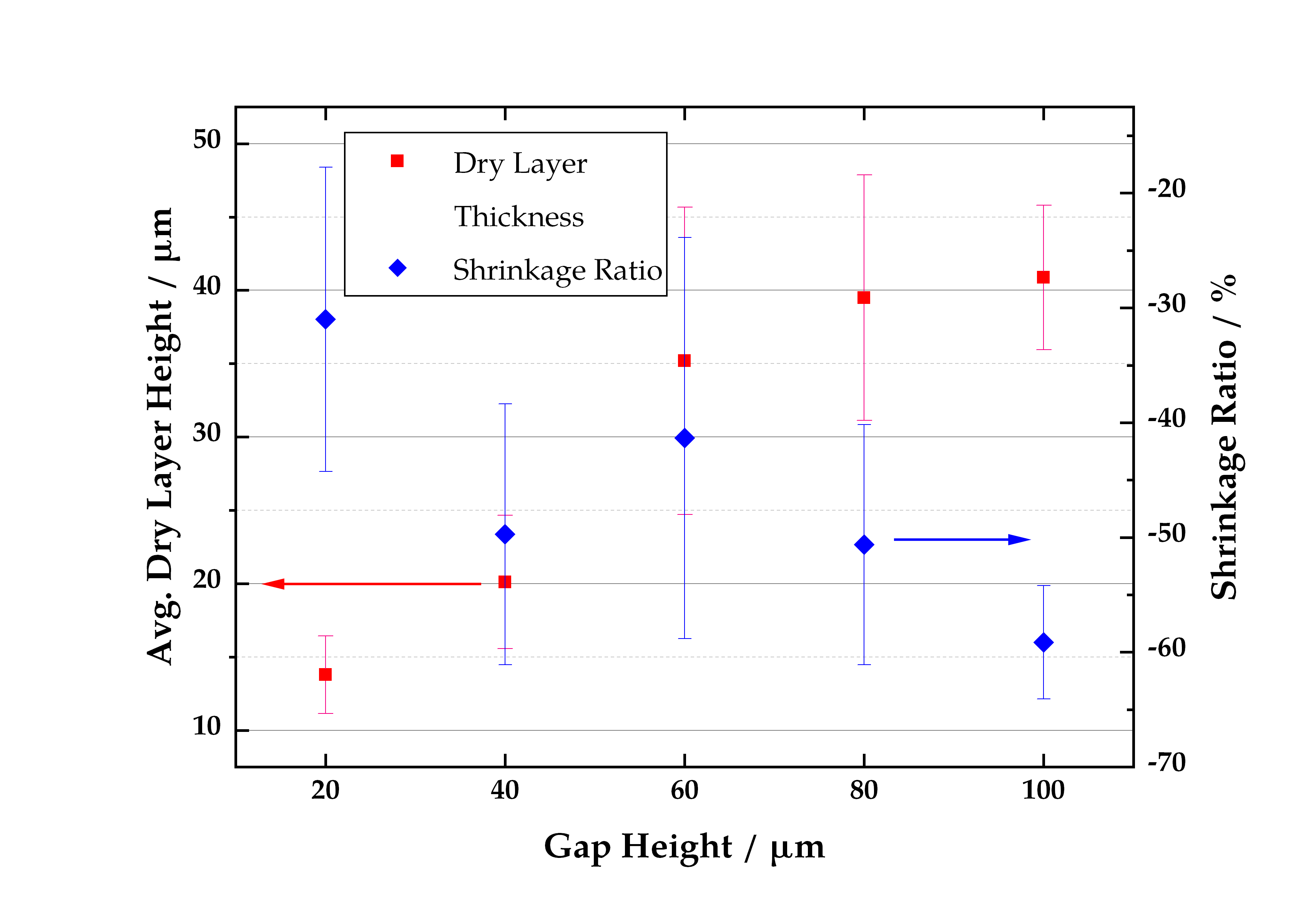


Figure S10. Average dry layer thickness and calculated shrinkage ratio of the coating compared to the initial doctor blade wet-gap setting.

## 4.11. Video of observing drying dynamics using thermal camera


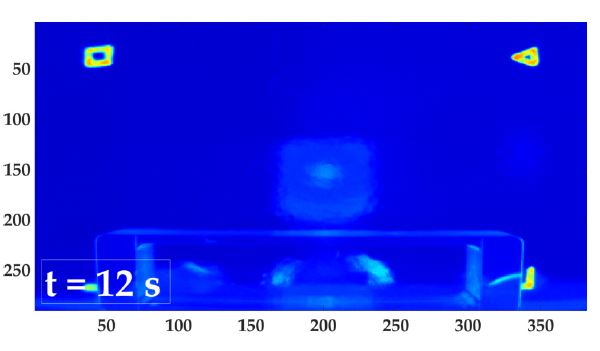


**b**


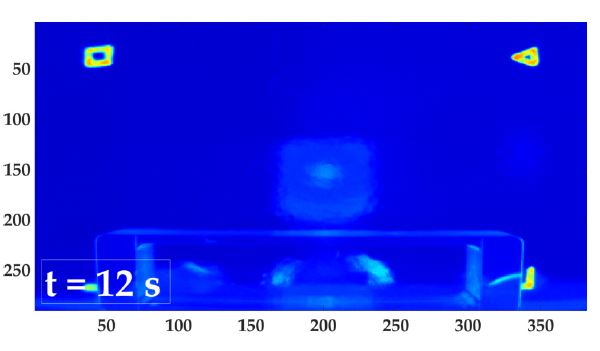


**a**

Video S1. Thermal video showing samples coated with different wet-gap heights using a doctor blade, with copper foil placed on a heated bed set to 60 °C. (a) 50 µm wet-gap, 60 °C bed temperature; (b) 100 µm wet-gap, 60 °C bed temperature. Note: Videos are available as separate files in the supporting information.

## 4.12. Defect formation during drying: thermal imaging observations

*
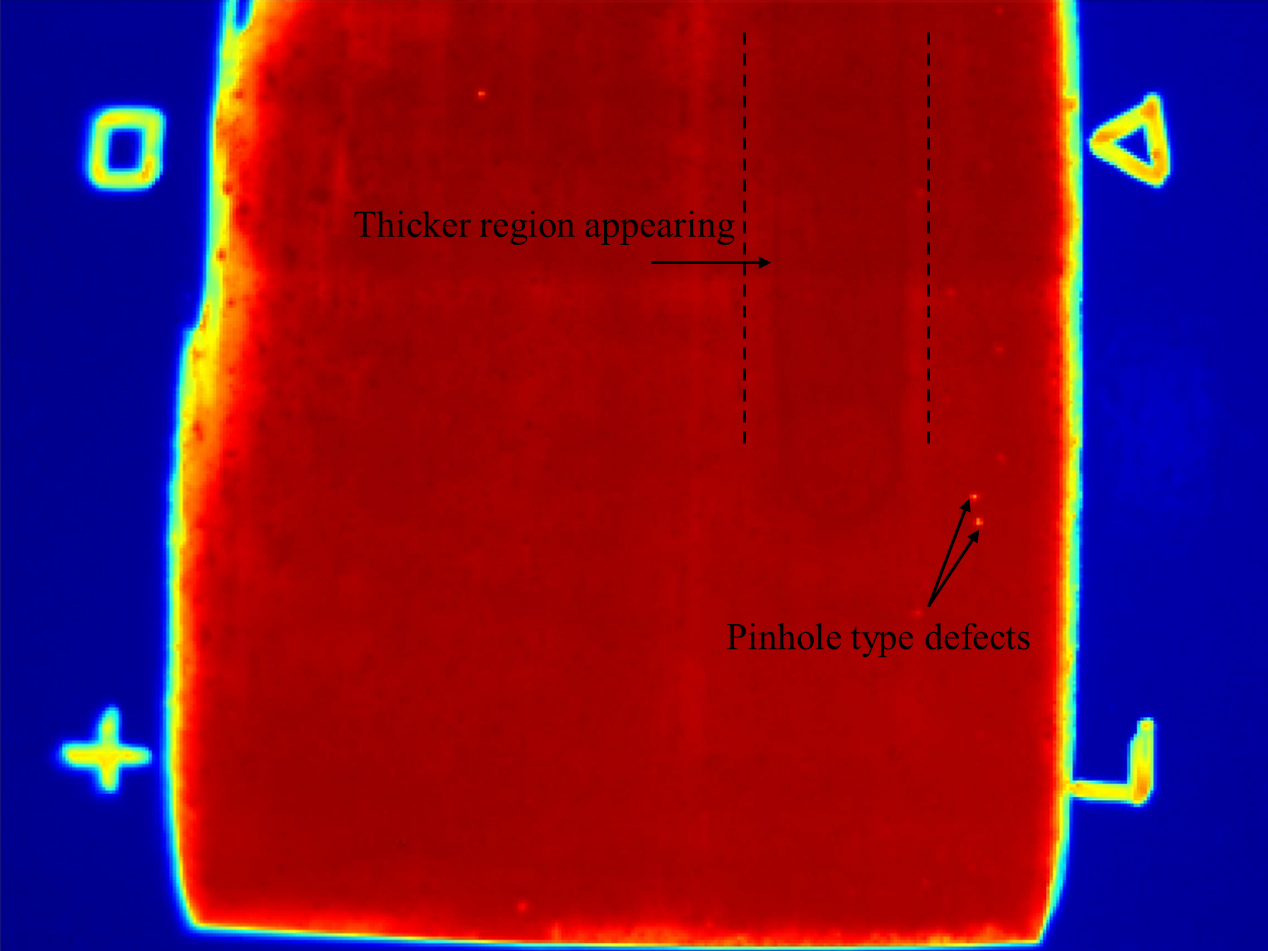
*

Figure S11. Thermal imaging snapshot (from video S1b) from the drying process at a **100 µm wet-gap and 60 °C**, showing the formation of pinhole-type defects (right) and a thicker region (top-right). The thicker region appears due to an initial slurry drop (unintentionally) before the main coating process, while pinhole defects become visible towards the end of the initial drying phase. These observations suggest that thermal imaging can capture defect formation dynamics during drying.

**References**

[1] A. Amin, M. Loewenich, S. O. Kilian, T. Wassmer, S. Bade, J. Lyubina, H. Wiggers, F. Özcan, D. Segets, *J. Electrochem. Soc.* **2023**, *170*, 20523.

[2] a) A. Du Baret de Limé, T. Lein, S. Maletti, K. Schmal, S. Reuber, C. Heubner, A. Michaelis, *Batteries & Supercaps.* **2022**, *5*; b) A. Schoo, R. Moschner, J. Hülsmann, A. Kwade, *Batteries.* **2023**, *9*, 111; c) T. Günther, D. Schreiner, A. Metkar, C. Meyer, A. Kwade, G. Reinhart, *Energy Tech.* **2020**, *8*; d) L. David, R. E. Ruther, D. Mohanty, H. M. Meyer, Y. Sheng, S. Kalnaus, C. Daniel, D. L. Wood, *Applied Energy.* **2018**, *231*, 446;

[3] Y. Liu, Y. Wang, J. Zhang, “New Machine Learning Algorithm: Random Forest”, in *Information Computing and Applications.* Springer Berlin Heidelberg; Imprint; Springer, Berlin, Heidelberg. **2012**, p. 246 ff.
